# Expression patterns of NKCC1 in neurons and non-neuronal cells during cortico-hippocampal development

**DOI:** 10.1093/cercor/bhac470

**Published:** 2022-12-27

**Authors:** Samu N Kurki, Pavel Uvarov, Alexey S Pospelov, Kalevi Trontti, Antje K Hübner, Rakenduvadhana Srinivasan, Masahiko Watanabe, Iiris Hovatta, Christian A Hübner, Kai Kaila, Mari A Virtanen

**Affiliations:** Molecular and Integrative Biosciences, University of Helsinki, 00014 Helsinki, Finland; Neuroscience Center, Helsinki Institute of Life Science, University of Helsinki, 00014 Helsinki, Finland; Molecular and Integrative Biosciences, University of Helsinki, 00014 Helsinki, Finland; Neuroscience Center, Helsinki Institute of Life Science, University of Helsinki, 00014 Helsinki, Finland; Molecular and Integrative Biosciences, University of Helsinki, 00014 Helsinki, Finland; Neuroscience Center, Helsinki Institute of Life Science, University of Helsinki, 00014 Helsinki, Finland; Neuroscience Center, Helsinki Institute of Life Science, University of Helsinki, 00014 Helsinki, Finland; SleepWell Research Program, Faculty of Medicine, University of Helsinki, 00014 Helsinki, Finland; Department of Psychology and Logopedics, University of Helsinki, 00014 Helsinki, Finland; Institute of Human Genetics, Jena University Hospital, Friedrich Schiller Universität, 07747 Jena, Germany; Molecular and Integrative Biosciences, University of Helsinki, 00014 Helsinki, Finland; Neuroscience Center, Helsinki Institute of Life Science, University of Helsinki, 00014 Helsinki, Finland; Department of Anatomy, Faculty of Medicine, Hokkaido University, Sapporo 060–8638, Japan; Neuroscience Center, Helsinki Institute of Life Science, University of Helsinki, 00014 Helsinki, Finland; SleepWell Research Program, Faculty of Medicine, University of Helsinki, 00014 Helsinki, Finland; Department of Psychology and Logopedics, University of Helsinki, 00014 Helsinki, Finland; Institute of Human Genetics, Jena University Hospital, Friedrich Schiller Universität, 07747 Jena, Germany; Molecular and Integrative Biosciences, University of Helsinki, 00014 Helsinki, Finland; Neuroscience Center, Helsinki Institute of Life Science, University of Helsinki, 00014 Helsinki, Finland; Molecular and Integrative Biosciences, University of Helsinki, 00014 Helsinki, Finland; Neuroscience Center, Helsinki Institute of Life Science, University of Helsinki, 00014 Helsinki, Finland

**Keywords:** NKCC1, Slc12a2, chloride regulation, bumetanide, neurodevelopmental disorders

## Abstract

The Na-K-2Cl cotransporter NKCC1 is widely expressed in cells within and outside the brain. However, our understanding of its roles in brain functions throughout development, as well as in neuropsychiatric and neurological disorders, has been severely hindered by the lack of reliable data on its developmental and (sub)cellular expression patterns. We provide here the first properly controlled analysis of NKCC1 protein expression in various cell types of the mouse brain using custom-made antibodies and an NKCC1 knock-out validated immunohistochemical procedure, with parallel data based on advanced mRNA approaches. NKCC1 protein and mRNA are expressed at remarkably high levels in oligodendrocytes. In immature neurons, NKCC1 protein was located in the somata, whereas in adult neurons, only NKCC1 mRNA could be clearly detected. NKCC1 immunoreactivity is also seen in microglia, astrocytes, developing pericytes, and in progenitor cells of the dentate gyrus. Finally, a differential expression of NKCC1 splice variants was observed, with NKCC1a predominating in non-neuronal cells and NKCC1b in neurons. Taken together, our data provide a cellular basis for understanding NKCC1 functions in the brain and enable the identification of major limitations and promises in the development of neuron-targeting NKCC1-blockers.

## Introduction

The Na-K-2Cl cotransporter, NKCC1, has wide expression patterns and numerous well-established functions, including cell division and migration, exocrine and endocrine secretion, immunological mechanisms, as well as ion and water homeostasis in various compartments of the organism (see references in Russell 2000; Pedersen et al. 2006; Wu and Sun 2015; Delpire and Gagnon 2018; Hung et al. 2018; Xu et al. 2021; Hartmann and Nothwang 2022). In contrast to this, there is little information on the developmental and cell-specific expression patterns of NKCC1 protein in brain parenchyma. Physiological and pharmacological experiments have definitely shown that NKCC1 is expressed in both immature and diseased central neurons (Yamada et al. 2004; Achilles et al. 2007; Sipila et al. 2009; Kaila, Price, et al. 2014; Blanquie et al. 2017; Sulis Sato et al. 2017; Kolbaev et al. 2020). However, the lack of valid immunohistochemical (IHC) procedures for reliable detection of NKCC1 in the brain has led to major confusions and controversies regarding, in particular, whether specific pharmacological targeting of neuronal NKCC1 by drugs such as bumetanide and its derivatives is possible (Löscher and Kaila 2022).

NKCC1 is expressed as two splice variants, NKCC1a and NKCC1b, which differ by alternative splicing of the exon-21, encoding 16 amino acids in the intracellular C-terminal domain of NKCC1a (Randall et al. 1997). It is likely that this difference at the protein level serves mainly the purpose of targeting and (sub)cellular localization, as exemplified by epithelial cells, in which the targeting of NKCC1a to the basolateral membrane depends on a dileucine motif encoded by exon-21 (Carmosino et al. 2008 but see also Koumangoye et al. 2019). Moreover, it has been suggested that the shorter variant, NKCC1b, is mainly expressed in the brain (Randall et al. 1997; Vibat et al. 2001).

The two main branches of NKCC1 research in the central nervous system (CNS) have thus far been focused on (i) the molecular mechanisms underlying the well-known “developmental GABA shift” that takes place during neuronal maturation and is tightly associated with an upregulation of the neuron-specific Cl^−^ extruder KCC2, which along with NKCC1 and seven other members belong to the family of Cation-Chloride Cotransporters (CCC) (Kaila, Price, et al. 2014). The shift from depolarizing to hyperpolarizing GABA action is attributable to the developmental upregulation of KCC2 (Rivera et al. 1999) but whether NKCC1 is up- or downregulated during neuronal maturation has been a matter of debate (see Virtanen et al. 2020). Notably, (ii) in diseased neurons, an opposite kind of shift is seen, from a hyperpolarizing to depolarizing action of GABA (Rivera et al. 2002; Huberfeld et al. 2007; Kaila, Ruusuvuori, et al. 2014). In numerous studies on neuronal development and disease, there have been attempts to estimate the relative contributions of KCC2 and NKCC1 based on western blots (WB) on brain tissue samples, an approach that is bound to fail because of the simple fact that KCC2 is a neuron-specific molecule while NKCC1 is not (for further Discussion, see Virtanen et al. 2020).

NKCC1 immunoreactivity (IR) has been reliably shown in various kinds of tissues and cell types outside the brain, e.g. the inner ear (Antoine et al. 2013), adrenal glands (Göppner et al. 2019), and dorsal root ganglia (Mao et al. 2012). In contrast, in work on the brain parenchyma, the lack of properly validated antibodies and IHC procedures has created a major obstacle for basic and translational research on NKCC1 gene regulation, functions, and pharmacology. Indeed, the previously published literature on the expression patterns of NKCC1 protein in the CNS shows highly divergent and mutually inconsistent results (for references, see Virtanen et al. 2020) and, to our best knowledge, none of these studies has met the rigorous criteria of validation by relevant knock-out (KO) control experiments (Fritschy 2008).

The present work is based on IHC experiments with two pan-NKCC1 antibodies (which detect both NKCC1a and NKCC1b) validated in NKCC1-KO brain sections, with extensive parallel data obtained by advanced RNA-based approaches (ultrasensitive RNAscope in situ hybridization, RNA-seq data analysis). We demonstrate here that during early postnatal development in mouse, NKCC1 protein can be detected in the somata of cortical and hippocampal pyramidal neurons. While neuronal NKCC1 mRNA expression continues into adulthood, the protein levels in mature neurons fall below our IHC detection. Moreover, we observed remarkably high levels of NKCC1 protein in oligodendrocytes (OLs) and readily detectable levels in microglia, astrocytes, developing pericytes, and dentate gyrus (DG) progenitor cells. Using advanced RNA methods and an NKCC1a splice variant selective, KO-controlled antibody, we further demonstrate that NKCC1a is predominant in glial cells, and NKCC1b in neurons. Our data as whole show that there is a developmental increase in the expression of NKCC1 in brain parenchyma, attributable to upregulation of the predominantly glial splice variant, NKCC1a. Thus, we provide here a molecular and cellular basis for novel experiments on NKCC1 functions in the CNS and for possible re-evaluation and reinterpretation of previous studies.

## Material and methods

### Animals

C57BL/6 J mice, NKCC1^+/+^, and NKCC1^−/−^ mice (Antoine et al. 2013) were housed in a conventional animal facility under a 12-h light–dark cycle (lights on between 6:00 am and 6:00 pm) and with food and water available ad libitum. The tissue samples were taken during the lights-on period, with animals of both sex randomly assigned to experimental conditions. The experiments were conducted according to the guidelines and with the approval of the National Animal Ethics Committee of Finland (Helsinki, Finland), the local Animal Ethics Committee of the University of Helsinki (Helsinki, Finland), and the local authorities of the state of Thuringia.

### RNA extraction from tissues and dissociated cultures

Adult mouse CNS regions (the most caudal part of the brainstem, cerebellum, the primary somatosensory cortex, hippocampus, olfactory bulb, the prefrontal cortex, spinal cord, optic nerve, and trigeminal nerve) and non-neural tissues (heart, kidney, liver, and testis) were dissected on ice and immediately frozen in liquid nitrogen. Mouse sensory cortex samples were also collected from newborn (P1), one week old (1 W), 2 W, 3 W, 1 month (1 M), and 3 M old mice. RNA isolation was done using the RNeasy Plus Mini kit (QIAGEN), which contains a genomic DNA removal step.

Primary cortical cultures were prepared from P0-P1 mouse pups as described previously (Beaudoin et al. 2012). Cortices were quickly dissected in ice-cold HBSS (Ca^2+^ and Mg^2+^ free) buffered with 10 mM HEPES (pH 7.3) and supplemented with 1 mM sodium pyruvate and 0.1% (wt/vol) glucose. Cells were dissociated by enzymatic treatment in 0.25% trypsin at 37 °C for 20 min. Mixed cultures (neurons + glia) were grown in 6-well plates (500,000 cells per well), coated with 0.01% poly-L-lysine (P4707, Sigma-Aldrich), and maintained in Neurobasal medium containing B-27 Supplement (17504044, ThermoFisher Scientific), 2 mM L-glutamine, and antibiotics (100 U/ml penicillin +10 μg/ml streptomycin). Pure glial cultures were prepared as described previously (Banker and Goslin, 1998) and maintained in DMEM containing 10% fetal bovine serum, 2 mM L-glutamine, and the antibiotics (100 U/ml penicillin +100 μg/ml streptomycin). No poly-L-lysine coating was applied for the wells used to grow pure glial cultures. After 14 days in vitro (div14), both types of cultures were briefly washed with ice-cold PBS, and total RNA was isolated using the RNeasy Plus Mini kit (QIAGEN), which contains a genomic DNA removal step.

Total RNA (400 ng for cultures and up to 1 μg for tissue samples) was reverse transcribed using Maxima First Strand cDNA Synthesis Kit for RT-qPCR (Thermo Fisher Scientific), which includes both oligo(dT) and random primers to provide an unbiased representation of 5′ and 3′ regions.

### RT-PCR analysis

Diluted (1:10) cDNA samples were used as templates in reverse transcription polymerase chain reaction (RT-PCR) (DreamTaq PCR Master Mix ThermoFisher Scientific) with primers amplifying the NKCC1a and NKCC1b variants (common primers): NKCC1-F 5′-ACCAAGGATGTGGTAGTAAATGTGG-3′ (exon-20) and NKCC1-R 5′-CAAGAAGCTTTTGGTCAGC-3′ (exon-22). PCR conditions included: 1 cycle of the initial denaturation at 95 °C for 3 min followed by 30 cycles at 95 °C for 20 s, 55 °C for 20 s, and 72 °C for 30 s. PCR reactions were resolved on 2% agarose gel.

The identity of the 220 bp heteroduplex was confirmed by gel-extraction and re-amplification with the common primers as described above. The re-amplification products were treated with S1 Nuclease (Thermo Fisher Scientific) according to the manufacturers’ recommendations. In tissue samples, NKCC1 mRNA levels were normalized to Ppib (5′-GGAGATGGCACAGGAGGAAA-3′, 5′-CCCGTAGTGCTTCAGCTTGAA-3′).

### Quantitative PCR analysis of the NKCC1a and NKCC1b mRNA splice variants

Diluted (1:10) cDNA samples were used as templates for quantitative real-time PCR (qPCR) analysis. Two sets of qPCR primers were designed to amplify specifically each of the NKCC1 splice variants (splice variant specific primers): NKCC1a-F 5′-ACCAAGGATGTGGTAGTAAATGTGG-3′, NKCC1a-R 5′-CCATCCTCTTCCTCATCTTTCTGTG-3′, NKCC1b-F 5′-ACCAAGGATGTGGTAGTAAATGTGG-3′, and NKCC1b-R 5′-GGGCCTTTGGATTCTTTCTGTG-3′. The specificity of each primer pair was tested using NKCC1a and NKCC1b templates and the following RT-PCR conditions: 1 cycle of the initial denaturation at 95 °C for 3 min followed by 40 cycles at 95 °C for 20 s, 66 °C for 20 s, and 72 °C for 20 s, with final amplification at 72 °C for 5 min.

The qPCR analysis was done using Maxima SYBR Green qPCR kit (Thermo Fisher Scientific). The reactions were run on CFX96 Real-Time PCR system (Bio-Rad) and analyzed using CFX Maestro software (Bio-Rad). PCR conditions were identical for the NKCC1a and NKCC1b mRNA variants (1 cycle: 95 °C 10 min, 40 cycles: 95 °C 15 s, 60 °C 1 min) with the subsequent melting curve step. Amplification efficiencies of the NKCC1a and NKCC1b primer sets were assessed using a standard curve with 5 serial dilutions of the adult mouse S1 cortex cDNA. Both primer pairs were designed to produce amplicons of about 100 bp, and both showed high amplification efficacy: 99.9% for NKCC1a and 98.6% for NKCC1b.

To assess the relative expression of the two splice variants, known amounts of NKCC1a and NKCC1b PCR templates were included in the qPCR analysis as six serial dilutions (4-fold each, the lowest 6th containing about 25,000 copies of the corresponding NKCC1 transcript per qPCR reaction). These values were used for constructing NKCC1a and NKCC1b standard curves for quantification of the absolute copy number in tissue samples.

### SDS-PAGE and immunoblotting

Sensory cortices were dissected from embryonic (E18.5), newborn (P0, day of birth), 1-week old (1 W), 2 W, 3 W, 1 month (1 M), 3M, 6M, and 1 year (1Y) old mice and homogenized on ice in RIPA lysis buffer (50 mM Tris·HCl, pH 8.0, 150 mM NaCl, 1% Triton X-100, 0.5% deoxycholic acid, and 0.1% SDS) supplemented with cOmplete™ Protease Inhibitor Cocktail (Roche Diagnostics). The lysates were precleared by centrifugation for 10 min (10,000 × g) at 4 °C, and protein concentrations of the supernatants were determined using DC Protein Assay Protein Assay Kit (Bio-Rad). Thirty micrograms of a total protein for each sample were mixed with 4x Laemmli sample buffer (Bio-Rad), incubated for 20 min at room temperature (RT), and run on a gradient 3–8% Tris-acetate SDS-PAGE (Bio-Rad). The lysates were not boiled before loading in order to avoid aggregation (see Results).

After running the SDS-PAGE, proteins were transferred from the gel onto Protran 0.45-μm nitrocellulose blotting membrane (Amersham), washed 3 times for 10 min at RT in TBST solution (Tris-buffered saline +0.1% Tween-20), blocked for 2 h at RT in the blocking solution (5% BSA in TBST), and incubated overnight at +4 °C with the RbC NKCC1 antibody (1:2,000 in 5% BSA in TBST).

Next day, membranes were washed 3 times for 10 min at RT in TBST and subsequently incubated for 2 h at RT with a donkey anti-rabbit HRP-linked antibody (NA9340, GE Healthcare), 1:2,000 dilution in the blocking solution. After 3 more washes in TBST solution, the membranes were imaged using Pierce ECL WB substrate (Thermo Fisher Scientific) and ChemiDoc MP imaging system (Bio-Rad). The accumulation mode was used to prevent signal overexposure and to keep measurements within the linear range of the camera’s sensitivity. Intensities of the bands were quantified using Image Lab software (Bio-Rad).

### RNAscope fluorescent in situ hybridization

The detection of NKCC1 and KCC2 mRNA by RNAscope kit (Advanced Cell Diagnostics) was done according to the supplier’s instructions, with minor modifications. P90 mice were terminally anesthetized by intraperitoneal injection of pentobarbital, followed by cardiac perfusion with ice-cold sterile PBS. Brains were immediately removed and frozen on dry ice. Coronal sections of 20 μm thickness were cut on a Leica CM1900 cryostat, mounted on glass slides (Super-FrostPlus; VWR International), and stored at −80 °C. Sections containing parietal and/or somatosensory cortex were chosen for analysis. Before hybridization, slices were postfixed in 4% paraformaldehyde (on ice) for 30 min. The specific NKCC1 and KCC2 in situ hybridization probes (Cat No. 311911-C2 and 311901, respectively) were provided by Advanced Cell Diagnostics. Slices were counterstained with 4,6-diamidino-2-phenylindole (DAPI, 1 μg/μl in PBS), mounted with ProLong Gold (Life Technologies), and stored at +4 °C until imaging.

### Quantitative analysis of RNAscope data

RNAscope sections were imaged on Zeiss Axio Imager.M2 light microscope equipped with ApoTome. The acquired images were then processed with a custom-written CellProfiler (Stirling et al. 2021) pipeline. First, masks for cells were created using IdentifyPrimaryObjects module on the DAPI channel followed by ExpandOrShrinkObjects module to account for cytoplasmic volume around the nucleus (radial expansion by 10 pixels, corresponding to 1.6 µm). KCC2 and NKCC1 mRNA speckles were identified using IdentifyPrimaryObjects and filtered by an intensity threshold set at a level which produced in sections stained with a negative control probe a single speckle per 10 cells (the acceptable level of unspecific background staining according to the RNAscope manual). Speckles were also filtered using a minimum area of 8 pixels to exclude detection artifacts which did not correspond to actual speckles in the raw data as judged by visual inspection. KCC2 and NKCC1 speckles were related to cell objects using RelateObjects module. A cell was classified as a neuron based on strong KCC2 mRNA expression, defined here as 10 or more KCC2 speckles per cell; or as a non-neuronal cell based on no KCC2 mRNA expression (zero KCC2 speckles). We analyzed 370 cortical cells classified as neurons and 413 cells in corpus callosum classified as non-neuronal. The data were collected from three animals, which were pooled due to low intersubject variability.

### Immunohistochemistry

Mice were terminally anesthetized by intraperitoneal injection of pentobarbital, followed by cardiac perfusion with 4% paraformaldehyde. Brains were removed, postfixed overnight in 4% paraformaldehyde, and embedded in paraffin. Coronal sections of 10–14 μm thickness were cut on Leica RM2255 microtome and mounted on glass slides. Sections containing parietal and/or somatosensory cortex were chosen for analysis.

Paraffin-embedded sections were deparaffinized in xylene, hydrated in a descending ethanol series (100, 94, 70, and 50%), and rinsed in MQ-water. For antigen retrieval, sections were boiled in 10 mM sodium citrate, 0.05% Tween 20 (pH 6.0) using a microwave oven (Bosch HTM742C) at 800 W for 3.5 min, followed by 90 W for 9 min. The beaker was kept at room temperature to cool down for 30–45 min. Sections were washed 10 min in PBS and permeabilized with 0.2% Triton-X-100 in PBS for 30 min, followed by a further epitope retrieval with 1% sodium dodecyl sulfate and 8% β-mercaptoethanol in PBS for 5 min. Sections were washed 4 × 10 min in PBS and incubated for 1 h in blocker solution containing 3% BSA, 0.3% Triton-X, and 10% goat serum in PBS. Primary antibodies were diluted in a modified blocker solution (1% BSA, 0.3% Triton-X, 1% goat serum in PBS) and applied to the sections overnight at +4 °C. Sections were then washed 3 × 10 min in PBS containing 0.2% Triton-X-100 and incubated for 2–4 h at room temperature with secondary antibodies in the modified blocker solution. After washing in PBS, nuclei were visualized with DAPI (1:2,000 in PBS); sections were washed again in PBS and mounted on glass slides with FluoromountG (ThermoFisher).

All primary and secondary antibodies and their dilutions are listed in Table 1. Anti-NKCC1 GpA antibody was produced against the amino acid residues 237–275 of mouse NKCC1 (NM_009194) using methods described elsewhere (Wei et al. 2013).

**Table 1:** List of antibodies used for IHC.

Primary antibodies	Host species	Dilution	Supplier and Cat. No
Anti-NKCC1, GpA (aa 237–275 of mouse NKCC1), affinity purified polyclonal	guinea pig	1:300 or 1:1000	Dr Masahiko Watanabe, custom-made
Anti-NKCC1, RbC (aa 1108–1141 of mouse NKCC1), affinity purified polyclonal	rabbit	1:300 or 1:1000	Dr Masahiko Watanabe, custom-made (Wei et al. 2013)
Anti-NKCC1a (aa 977–991 of mouse NKCC1), serum	rabbit	1:300 or 1:1000	Dr Christian Hübner, custom-made (Göppner et al. 2019)
Anti-NKCC1a (aa 977–991 of mouse NKCC1), affinity purified polyclonal Anti-beta-IV-spectrin	rabbit rabbit	1:300 or 1:1000 1:500	Dr Christian Hübner, custom-made (Göppner et al. 2019) Dr Matthew N. Rasband, custom-made (Yang et al. 2004)
Anti-CD13, polyclonal	goat	1:500	AF2335, R&D Systems
Anti-CNPase, monoclonal	mouse	1:2000 or 1:5000	AMAb91072, Atlas Antibodies
Anti-DCX, polyclonal	chicken	1:200 or 1:500	Ab153668, Abcam
Anti-GFAP, affinity purified polyclonal	chicken	1:500	Ab5541, EMD Millipore Corp.
Anti-Iba1, polyclonal	rabbit	1:500	019–19,741, Fujifilm Wako
Anti-MAP2, polyclonal	rabbit	1:250	Ab5622, EMD Millipore Corp.
Anti-MBP, polyclonal	chicken	1:5000	PA1–10008, Invitrogen
Anti-Olig2, polyclonal	goat	1:200	AF2418, R&D Systems
Anti-PDGFRalpha (D1E1E), monoclonal	rabbit	1:500	#3174, Cell Signaling Technology
Anti-VGAT, polyclonal	rabbit	1:1000	131–002, Synaptic Systems
Anti-VGLUT1, polyclonal	guinea pig	1:3000	Ab5905, EMD Millipore Corp.
Anti-VGLUT2, polyclonal	guinea pig	1:300	135–404, Synaptic Systems
Secondary antibodies	Host species	Dilution	Supplier and Cat. No
Anti-chicken-Alexa633	goat	1:1000	A21103, Invitrogen
Anti-guinea pig-Alexa488	goat	1:1000	Ab150185, Abcam
Anti-guinea pig-Alexa568	goat	1:1000	A11075, Invitrogen
Anti-mouse-Alexa488	goat	1:1000	A11001, Life Technologies
Anti-rabbit-Alexa488	goat	1:1000	A11034, Life Technologies
Anti-rabbit-Alexa568	goat	1:1000	A11036, Life Technologies
Anti-goat-Alexa488	donkey	1:1000	Ab150129, Abcam
Anti-rabbit-Alexa568	donkey	1:1000	A10042, Life Technologies

### Image acquisition and analysis

Epifluorescent images were collected with Zeiss Axio Imager.M2 light microscope equipped with ApoTome, using 20X and 40X/oil immersion objectives (0.8 NA and 1.3 NA, respectively), and Leica DM6000B light microscope, using 20X immersion glycerol objective (0.7 NA). Confocal images were taken with Leica Stellaris 8 FALCON confocal microscope, using 20X and 40X/glycerol immersion objectives (0.75 NA and 1.25 NA, respectively). The pinhole was set at 1 Airy unit and wavelengths were separated to sequentially image channels using the full intensity range of detection.

For comparison of NKCC1 IR in WT and NKCC1^−/−^ mice, samples were processed in parallel and imaged with identical settings. All parameters for offline image processing were identical between the genotypes. The genotype of animals was strictly blinded to the investigators until all images had been processed offline.

Images were processed using Zen 3.1 (Carl Zeiss Microscopy GmbH) and LAS X 3.5.6 (Leica Microsystems GmbH). Signals in two channels were tentatively considered to be colocalized if the area with highest intensity of the local signal in one channel overlaps that of the other channel. Colocalization was confirmed with Imaris software (version 9.7.2, Bitplane) using Section View to simultaneously view the orthogonal XY, XZ, and YZ-planes of the area of interest in cell types which had spatially restricted patterns of NKCC1 IR.

The quantification of astrocytic NKCC1 IR was done using confocal stacks. Astrocytes were first identified in maximum projection images based on the immunoreactivity for both GFAP and S100B. NKCC1 IR was then analyzed from single Z-planes, and an astrocyte was counted as NKCC1-positive if it showed in the soma and/or proximal rami clear NKCC1 IR that was above the IR of the surrounding tissue. We analyzed 11–12 astrocytes per animal, from three animals.

Microglial NKCC1 expression was quantified in a similar manner, using IBA1 for microglial detection. We analyzed 31–55 microglia per animal, from three animals.

### Analysis of RNA-seq databases

Mouse *Slc12a2* gene expression in different cell types was analyzed by retrieving data from four openly available single-cell RNA-seq databases (Habib et al., https://singlecell.broadinstitute.org/single_cell/study/SCP1/-single-nucleus-rna-seq-of-cell-diversity-in-the-adult-mouse-hippocampus-snuc-seq; Saunders et al., Gene Expression Omnibus (GEO) repository accession “GSE116470”; Zeisel et al., http://loom.linnarssonlab.org/; Loo et al., GEO accession “GSE123335”). Cells were classified into different cell types using annotation tables provided by each of the databases, except for Zeisel et al., in which the data were already segregated by cell type at the repository level. To achieve quantitatively comparable measures for *Slc12a2* expression, we transformed all datasets into ln(TPM + 1) scale, by normalizing the *Slc12a2* unique molecular identifier (UMI) counts of each cell by the total number of UMIs observed in that cell, multiplying this ratio by 10^6^ to obtain TPM and finally taking ln(TPM + 1). The analysis was conducted at the level of individual cells in all studies except for Saunders et al., which provided the data grouped into subclusters, each of which represents a specific cell type.

We did not set any additional detection limits beyond what was used in the respective original study: Habib et al. only considered genes detected if their transformed expression levels were equal to or above 3 (1.1 in ln(TPM + 1) scale), whereas in all the other studies, even a single detected *Slc12a2* transcript was counted in.

Data were processed in Microsoft Excel, except for those in Zeisel et al., in which a custom-written Python code (Python 3 Reference Manual) was used to handle the “loom” -file type (http://linnarssonlab.org/loompy/index.html).

To quantify the expression of NKCC1a and NKCC1b splice variants in neurons, mature OLs, and astrocytes, we analyzed cell-type-specific RNA-sequencing data of Zhang et al. (2014; GEO data set GSE52564). We aligned the sequence reads to mouse genome GRCm38 using STAR aligner v2.5.4b (Dobin et al. 2012) and visualized sequences overlapping *Slc12a2* (NKCC1) with IGV v2.5.2 sashimi plot (Broad institute) to quantify number of intron splice junctions that skip exon-21 relative to those containing it.

### Statistics

Unless otherwise indicated, 3–4 mice were used for each condition. In RNA-seq analysis, the experimental unit was the cell or the enriched cell sample.

Statistics were calculated with GraphPad Prism (version 8, La Jolla, CA). All values are presented as the mean ± the standard deviation (SD), unless otherwise indicated. Normality was tested for each distribution (Shapiro–Wilk normality test). For multiple comparisons of Gaussian distributions, statistical significance was determined using one-way ANOVA followed by Bonferroni’s or Tuckey’s post hoc tests (corrected for multiple comparisons). For all tests, α was set to 5% and *P* < 0.05 was considered as statistically significant.

## Results

In the following, we will first describe the cellular expression pattern of NKCC1 protein and mRNA in the adult mouse brain parenchyma. Thereafter, we focus on the differential expression of the NKCC1 splice variants in neurons and non-neuronal cells. Finally, we present data on the developmental expression patterns of NKCC1 in specific cell types in the postnatal mouse brain.

### NKCC1 protein is abundant in OLs and microglia, and detectable in astrocytes but not in neurons of the adult mouse cerebral cortex

**Fig. 1:**
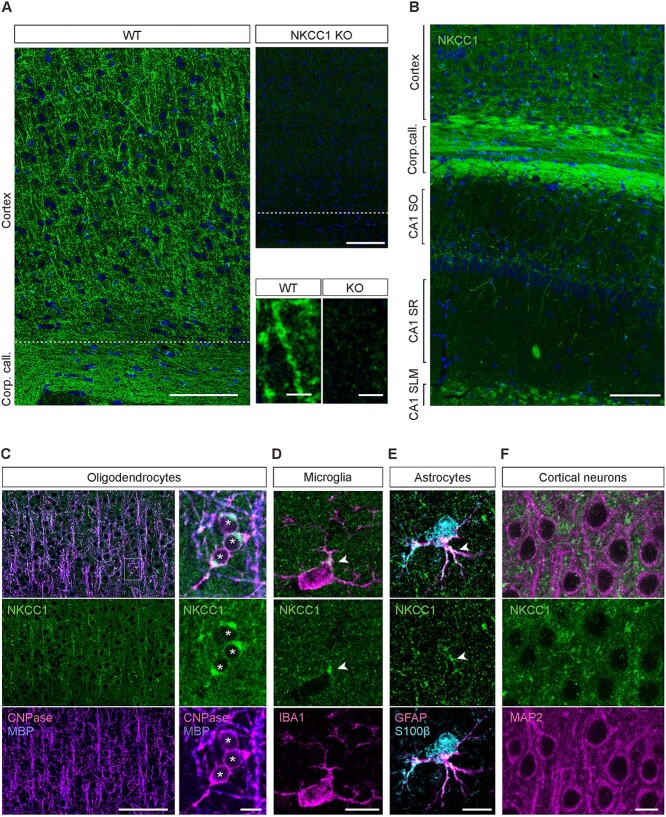
NKCC1 protein is strongly expressed in glial cells of the adult mouse cortex and hippocampus. (A) NKCC1 immunoreactivity in the somatosensory cortex of WT mice. Sections from NKCC1 KO mice show a weak and homogenous background signal. Identical results were obtained with both GpA and RbC NKCC1 antibodies (*n* = 4 WT/KO pairs). Higher magnification images are shown in the bottom right corner. (B) Overview of the NKCC1 expression in the cortex and hippocampus. (C) The strong cortical and callosal NKCC1 protein signal mostly originates from oligodendrocytes, colocalizing with MBP and the oligodendrocytic marker CNPase. (D) Microglial cells, detected by their Iba1 expression, showed one or few clusters of NKCC1 IR in the soma, often close to a ramification (arrowhead). (E) Astrocytes, identified by expression of both GFAP and S100B, showed NKCC1 expression in some of their rami (arrowheads). (F) Cortical neurons identified by MAP2 IR do not express detectable levels of NKCC1 in the somatodendritic compartment. Scale bars A: 100 μm (top row pair) and 5 μm (bottom row pair), B: 100 μm, C: 100 μm (left) and 10 μm (right), D–F: 10 μm.

We first tested the specificity of several NKCC1 antibodies and optimized the IHC protocol using sections from adult wild-type (WT) and NKCC1 KO (NKCC1^−/−^) mice (Fig. 1A; Supplementary Fig. 1A and B). While strong NKCC1 IR was readily detectable in the choroid plexus with all IHC protocols and NKCC1 antibodies tested, the specific signal in the brain parenchyma could only be seen after strong epitope retrieval. In both paraffin and cryosections, an epitope retrieval step in SDS and β-mercaptoethanol (Supplementary Fig. 2; see Material and methods for details) was necessary for obtaining NKCC1 IR. Both pan-NKCC1 antibodies gave an essentially identical staining pattern in the WT sections, and this pattern was completely absent in the KO sections.

In adult WT mice, we observed prominent NKCC1 IR in the corpus callosum and NKCC1-positive cellular processes reminiscent of neurites in the cortex and hippocampus (Fig. 1B). In addition to this, a more homogenous granular background was visible across the cortex and hippocampus (see Discussion). Notably, a similar background pattern was seen with both NKCC1 antibodies (GpA and RbC). In contrast, only a very weak background signal with no pattern was observed with both antibodies in sections from NKCC1 KO mice. Together with the fact that the two antibodies used presently target different regions of the NKCC1 protein (Supplementary Fig. 1A), this indicates that the more diffuse signal is specific and likely arises from NKCC1-containing intermingled and probably partly superimposed elements of various cellular origins.

In order to identify the cells generating the distinct patterns of NKCC1 IR, we conducted a series of double and triple stainings with several cell-type markers. The most prominent NKCC1 IR present in the corpus callosum, cortex, and hippocampus showed remarkable colocalization with the myelin basic protein (MBP) and the oligodendrocyte (OL) marker CNPase, with nearly all of the detectable NKCC1 IR colocalizing with the OL markers and nearly all myelinated fibers expressing NKCC1 (Fig. 1C, for hippocampal expression patterns see Supplementary Fig. 3). The OL somata also showed strong NKCC1 IR, with part of the signal most likely originating from the intracellular compartment. Our results agree with the existing evidence on the various roles of NKCC1 in the OL lineage (Wang et al. 2003; Chen et al. 2007; Wu and Sun 2015).

Microglia in vivo have recently been shown to express NKCC1 protein (Tóth et al. 2022), and we confirmed these results in the present study based on co-IR with the microglial marker Iba1 (Fig. 1D, Supplementary Fig. 4). We observed one or a few clearly outlined NKCC1 positive puncta located in the soma or the proximal parts of the ramifications in 84 ± 6% of microglia. However, with our current resolution, we cannot distinguish with certainty whether these NKCC1 clusters are intracellular and/or on the plasma membrane.

In line with functional evidence indicating the presence of NKCC1 in astrocytes (Pedersen et al. 2006; Larsen et al. 2014; Henneberger et al. 2020), we found NKCC1 IR colocalizing with cells identified as astrocytes by expression of molecular markers GFAP and S100B. The NKCC1 IR in these cells was variable, dispersed on somata and/or ramifications. However, it was quite consistently detectable: in 85 ± 11% of astrocytes (Fig. 1E, Supplementary Fig. 5).

We obtained negative results in colocalization experiments with the neuronal marker MAP2 (Fig. 1F), the pericytic marker CD13, the oligodendrocyte precursor marker PDGFRα, and the presynaptic markers for glutamatergic terminals VGLUT1 and VGLUT2 (Supplementary Figs. 6 and 7A and B). In experiments with VGAT (the presynaptic marker for GABAergic terminals), the interpretation of colocalization was more ambiguous and did not pass our preset criteria for a robust positive finding (Supplementary Fig. 7C, see Discussion).

### NKCC1 mRNA is present in neurons of the adult cortex and hippocampus

**Fig. 2:**
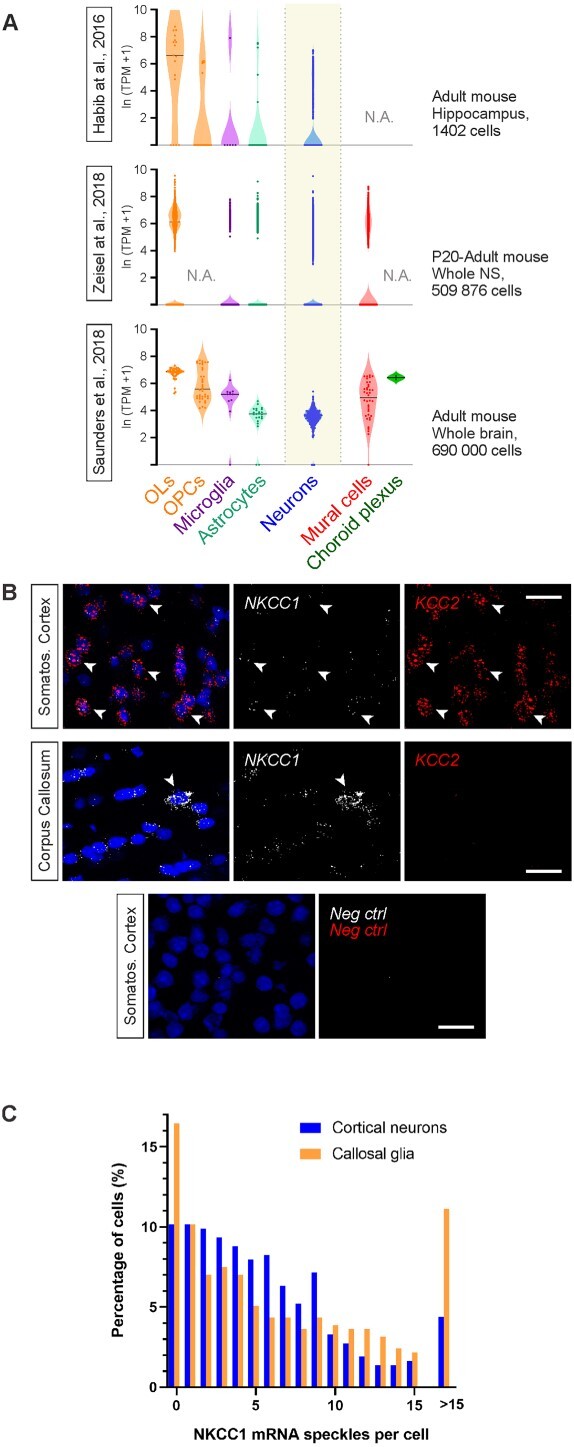
NKCC1 mRNA is expressed in non-neuronal cells as well as in adult cortical and hippocampal neurons. (A) Single-cell RNA-seq analysis shows highest NKCC1 mRNA levels in oligodendrocytes, but clear expression can also be detected in neurons. Expression of the NKCC1-encoding *Slc12a2* gene was analyzed in three independent datasets (Habib et al. 2016; Saunders et al. 2018; Zeisel et al. 2018). NS = Nervous system. (B) Ultrasensitive RNA in situ hybridization (RNAscope) shows very high expression of NKCC1 in a subset of non-neuronal cells, as exemplified in the corpus callosum (arrowhead). Neurons in the somatosensory cortex (arrows) express high levels of KCC2 (red) and show weak to moderate NKCC1 mRNA expression. Negative control probes give no detectable signal in the cortex, confirming the specificity of the RNAscope approach. (C) Quantification of RNAscope experiment demonstrates that NKCC1 mRNA can be detected in ~90% of cortical neurons (layer 5 examined here) identified by strong KCC2 mRNA expression. Some glial cells show a very high level (>15 speckles) of NKCC1 mRNA, and these cells are densely present in corpus callosum, suggesting that they are OLs. Scale bars 20 μm.

The lack of detectable neuronal NKCC1 IR in adult animals prompted us to ask whether neurons in the heathy adult brain (cf. Introduction) might express NKCC1 mRNA. First, we analyzed *Slc12a2* gene expression in three mouse single-cell RNA-seq databases (Habib et al. 2016; Saunders et al. 2018; Zeisel et al. 2018). Interestingly, all three databases showed clear NKCC1 mRNA expression in neurons. However, the consistently strongest expression of NKCC1 mRNA is in OLs, which is in line with our IHC protein analyses (Fig. 2A).

To further test whether neurons express NKCC1 mRNA, we used the ultrasensitive multiplex fluorescent in situ hybridization approach (RNAscope), which gives a semi-quantitative measure of mRNA expression in fixed brain slices (Wang et al. 2014). As a neuronal marker, we used KCC2, which is expressed in all CNS neurons in the adult, with the exception of a few neuronal subpopulations in the thalamus, hypothalamus, and substantia nigra (Kanaka et al. 2001; Gulacsi et al. 2003; Bartho et al. 2004).

In sections from adult mice (P90), a strong KCC2 mRNA signal was detected in regions with high density of neuronal cell bodies as described before (Rivera et al. 1999), such as cortical layers 2–6, and hippocampal pyramidal cell layer (Supplementary Fig. 8A and B). As expected, only a few cells in cortical layer 1 and the corpus callosum expressed KCC2 mRNA (cf. von Engelhardt et al. 2011), and no KCC2 mRNA signal was detected in the choroid plexus epithelium (Supplementary Fig. 8C).

In most neurons (identified on the basis of KCC2, see Methods) in the adult primary somatosensory cortex (S1) and hippocampus, RNAscope demonstrated moderate (5–10 speckles per cell) to low (<5 speckles) NKCC1 mRNA levels (Fig. 2B and C). Notably, in ~90% of cortical layer 5 neurons at least one NKCC1 mRNA speckle was detected by RNAscope (Fig. 2C). In line with the above data on cell-type-specific NKCC1 protein expression, NKCC1 mRNA expression was highly variable in KCC2-negative, non-neuronal cells.

Some non-neuronal cells had very high NKCC1 mRNA levels (>15 speckles per cell). These cells were scattered across all cortical layers and the hippocampus, with highest density in the corpus callosum (11% of cells showing >15 speckles) (Fig. 2B and C, Supplementary Fig. 8A), suggesting that these cells might be OLs.

RNAscope demonstrated very high NKCC1 mRNA levels (>15 speckles per cell) in the epithelial cells of the choroid plexus (Supplementary Fig. 8C), where NKCC1 is known to be strongly expressed. Negative control probes produced no signal, confirming the specificity and sensitivity of the ultrasensitive RNAscope approach (Supplementary Fig. 8).

### NKCC1a and NKCC1b are the major glial and neuronal mRNA splice variants, respectively

**Fig. 3:**
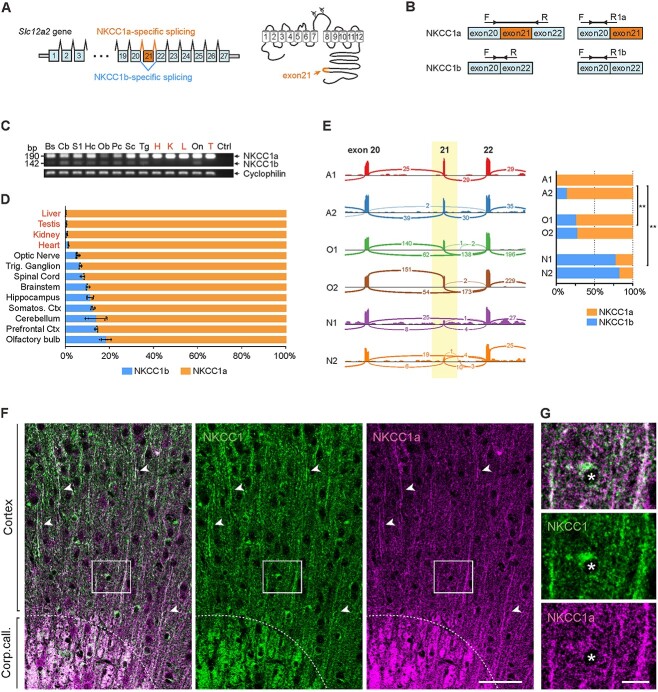
NKCC1a is the major splice variant in the adult mouse brain, with preferential expression in glia, whereas NKCC1b is preferentially expressed in neurons. (A) Gene model of *Slc12a2* and schematic topology of NKCC1. NKCC1a contains the exon-21 in the intracellular C-terminal domain, whereas in NKCC1b, it is spliced out. (B) Design of the common primers (left) and splice variant specific qPCR primers (right). (C) In adult mice, NKCC1a mRNA is expressed in all tissue types examined, whereas NKCC1b mRNA expression is limited to neuronal tissues. Cyclophilin B was used as a normalization control. The experiment was repeated with four mice, all giving a similar result. *Bs: brainstem, Cb: cerebellum, S1: primary somatosensory cortex, Hc: hippocampus, Ob: olfactory bulb, Pc: prefrontal cortex, Sc: spinal cord, Tg: trigeminal ganglion, On: optic nerve, H: heart, K: kidney, L: liver, T: testis.* (D) Quantification of the relative expression of splice variants measured by qPCR in neuronal and non-neuronal tissues. (E) *Left panel:* Single-cell RNA sequencing shows alternative splicing of NKCC1 in neuronal and non-neuronal cells. Sashimi plot showing splice junction reads from aligned RNA-seq data in different cell types. The thickness of each curved line corresponds to the number (as illustrated) of mRNA molecules spliced from one exon to another. In neurons, most of the mRNA reads are spliced directly from exon-20 to exon-22, whereas in oligodendrocytes and in astrocytes, exon-21 is preferentially included. Two independent samples, each containing specifically enriched preparations of the analyzed cell type, were analyzed separately (A1 and A2 for astrocytes, O1 and O2 for myelinating oligodendrocytes, N1 and N2 for neuronal samples). *Right panel:* Relative expression of NKCC1a and NKCC1b splice variants in the enriched astrocyte, oligodendrocyte, and neuronal samples (One-way ANOVA, with Tukey multiple comparison of means, ^*^^*^: *P* < 0.01). (F) Comparison of panNKCC1 (GpA) vs. NKCC1a-specific immunoreactivity, in the cortex and corpus callosum (above and below the dotted line, respectively). Arrowheads indicate individual myelinated fibers that show high IR with both antibodies. (G) Close-up showing an individual OL soma (indicated by the asterisk) in the region denoted by a rectangle in F. NKCC1a-specific IR is less pronounced in the OL somata compared to the myelinated fibers, whereas the panNKCC1 antibody gives a stronger signal in the OL somata. Scale bars F: 50 μm, G: 10 μm.

The existence of the two NKCC1 splice variants (Fig. 3A) has long been acknowledged (Randall et al. 1997). As compared with other tissues, NKCC1b is preferentially expressed in the brain (Randall et al. 1997; Vibat et al. 2001), but little is known about the cell-type specificity of its expression. To test whether NKCC1b might be a neuronal splice variant, we designed common PCR primers for simultaneous amplification of both splice variants (semiquantitative RT-PCR) as well as variant specific primers for the qPCR (Fig. 3B). The specificity of qPCR variant specific primers was tested using DNA constructs encoding NKCC1a or NKCC1b as PCR templates. As expected, NKCC1a primers could only amplify the NKCC1a variant, while the NKCC1b primers specifically recognize NKCC1b (Supplementary Fig. 9A). The common PCR primers gave products corresponding to the NKCC1a (190 bp) and NKCC1b (142 bp) variants. Furthermore, we occasionally detected a third PCR product (~220 bp) that corresponds to the heterodimer of the NKCC1a and NKCC1b single strands amplified in the same PCR reaction (Supplementary Fig. 9B and C). S1 nuclease treatment (cleaves single-stranded but not double-stranded DNA) eliminated the NKCC1a/NKCC1b hybrid band in the agarose gel (Supplementary Fig. 9D).

PCR with the common primers showed that both NKCC1a and NKCC1b variants were present in all neural samples (brain and spinal cord of adult mice). In contrast, non-neural samples (heart, kidney, liver, and testis) showed strong expression of the NKCC1a variant, while NKCC1b was hardly detectable (Fig. 3C). Notably, both variants were clearly detected in the optic nerve, which is in fact a pure white-matter tract (not a conventional “nerve”), consisting of retinal ganglion cell axons and oligodendrocytes.

Next, we quantified the relative contribution of the NKCC1b splice variant to the total NKCC1 mRNA expression in the neural samples using qPCR. In brain and spinal cord samples, NKCC1b variant comprised on average ~10% of the total NKCC1 mRNA expression, while in the non-neural samples, this was only ~1–2% (Fig. 3D). Similar results were obtained comparing the NKCC1 splice variant expression in vitro in primary dissociated cultures of rat cortical neurons. NKCC1a was the only variant expressed in pure glial cultures, whereas in mixed neuronal and glial cultures, both NKCC1 variants were detected (Supplementary Fig. 10). Taken together, these data suggest that while NKCC1a is preferentially expressed in glia, NKCC1b is the major neuronal splice variant.

We then analyzed RNA splicing in neurons, OLs, and astrocytes in the RNA-Sequencing Transcriptome and Splicing Database (Zhang et al. 2014). Individual RNA-seq reads were aligned with the genomic coordinates of the mouse *Slc12a2* exon-intron model, allowing the unambiguous identification of the NKCC1 mRNA splicing events occurring in each analyzed cell type (Fig. 3E). In neurons, a majority (76%, 44 out of 58) of the RNA-seq reads lacked the exon-21 sequence and thus corresponded to the NKCC1b splice variant, while a minority (24%, 14 out of 58) represented the NKCC1a variant. In contrast, in OLs, the proportions were reversed, with 71% containing NKCC1a reads (291 out of 407) and 29% NKCC1b reads (116 out of 407). In astrocytes, the relative expression of NKCC1a was even higher, with 97% of all reads corresponding to the NKCC1a variant (64 out of 66). These results indicate that NKCC1a mRNA is the major NKCC1 variant in OLs and astrocytes, while NKCC1b mRNA is the main one in neurons.

### Differential expression patterns of NKCC1a protein

In order to examine whether the cell-type-specific mRNA expression patterns of NKCC1a and NKCC1b are reflected at the level of the two proteins, we used a unique NKCC1a-selective antibody generated against the exon-21 encoded region (Göppner et al. 2019). We first validated the specificity of the NKCC1a-selective antibody in brain tissue from WT and KO animals and confirmed that panNKCC1 antibodies recognized both variants (Supplementary Fig. 1C and D). In excellent agreement with our mRNA expression data, NKCC1a protein could be detected in the myelinated fibers, indicating the presence of NKCC1a protein in OLs (Fig. 3F). To our surprise, in contrast to the panNKCC1 antibodies (GpA and RbC), the NKCC1a-specific antibody gave a much weaker signal in the somata of OLs than in the myelinated fibers (Fig. 3G, see Discussion).

### The upregulation of NKCC1 in the postnatal mouse cortex is due to an increase in the expression of the main glial variant, NKCC1a

**Fig. 4:**
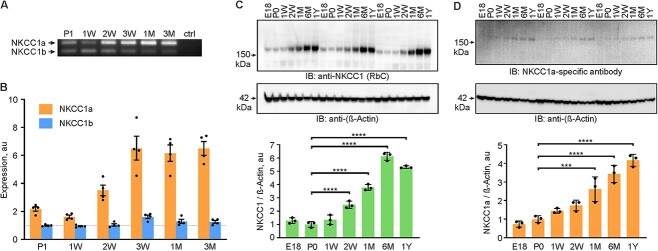
The developmental upregulation of NKCC1 in the mouse neocortex is due to an increase in the expression of the non-neuronal splice variant. (A) Both NKCC1 splice variants can be detected by RT-PCR throughout postnatal development. (B) Real-time qPCR data show that NKCC1a mRNA is steeply upregulated between the first and second postnatal weeks of the cortical development, while NKCC1b expression stays relatively stable. Values are normalized to the average NKCC1b expression at P1. (*n* = 4 for each group; One-way ANOVA, with Bonferroni’s multiple comparison of means; NKCC1a: P1–P21 *P* < 0.0001, P1–P30 *P* = 0.0001, P1–P90 *P* < 0.0001; NKCC1b: P1–P21 *P* = 0.0015). (C) NKCC1 protein levels increase several fold during the first six postnatal months. (D) NKCC1a protein expression levels increases over 4-fold during the first postnatal year. (*n* = 3 for each group; One-way ANOVA, with Bonferroni’s multiple comparison of means; ^*^^*^^*^^*^: *P* < 0.0001).

Next, we asked how the expression of the two NKCC1 splice variants evolves in the cerebral cortex during postnatal development. NKCC1a mRNA, which is mainly expressed by glial cells, showed a robust 4-fold upregulation within the second and third postnatal weeks (Fig. 4A and B). In contrast, NKCC1b mRNA, which is preferentially expressed in neurons, remained at a nearly constant level during the first three postnatal months, with a slight transient increase around the postnatal week 3. Thus, the total NKCC1 mRNA expression in the cerebral cortex increases with age, and this increase is mainly due to the expression of NKCC1a.

To determine whether and how the developmental profile of NKCC1 protein expression is related to the total NKCC1 mRNA profile, we used WB with a pan-NKCC1 antibody (RbC). First, we validated the approach by using cortical lysates from WT and NKCC1^−/−^ mice (Supplementary Fig. 11). Importantly, we noticed that lysate heating should be avoided, since it results in NKCC1 aggregation and subsequent fading of the monomeric band in WB (Supplementary Fig. 11), similar to what has previously been reported for KCC2 (Blaesse et al. 2006; Supplementary Fig. 3 in Markkanen et al. 2017). Analysis of the cortical lysates in a wide developmental time window, spanning from embryonic day 18 (E18) to 1 year, revealed a marked increase in total NKCC1 protein expression (Fig. 4C). The steepest phase of NKCC1 protein upregulation occurred during the first postnatal month with the NKCC1 protein levels increasing more than 3-fold compared with P0. This was followed by a further 1.6-fold increase until a plateau was reached at around 6 months of age. Furthermore, WB with the NKCC1a-specific antibody in the same cortical samples showed that the total expression of the non-neuronal variant increased over 4-fold during the first postnatal year (Fig. 4D).

Together, these results indicate that NKCC1 is upregulated during postnatal development and that this is attributable to an increase in the expression of the glial NKCC1a splice variant.

### In the perinatal cortex and hippocampus, NKCC1 mRNA and protein are strongly expressed in neurons, microglia, and blood vessels

**Fig. 5:**
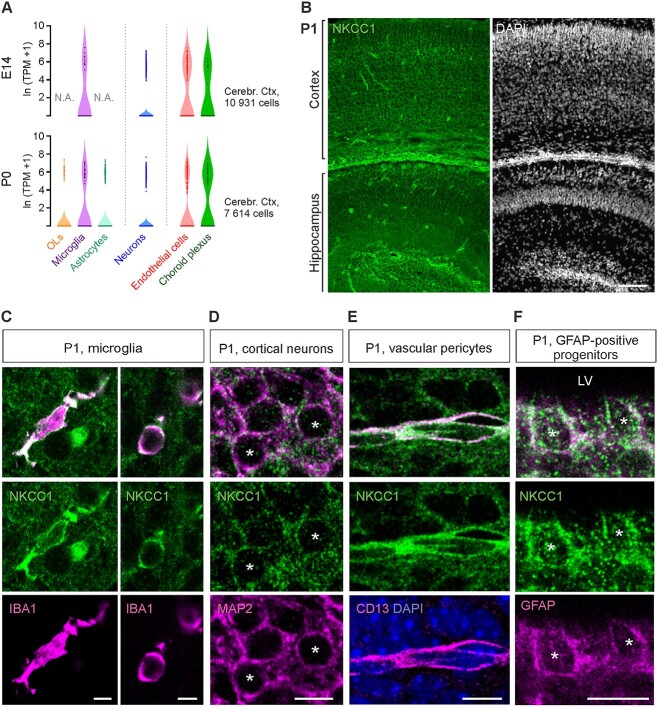
In the P1 cortex and hippocampus, NKCC1 is strongly expressed in microglia, blood vessels, progenitor cells, and neurons. (A) scRNA-seq analysis indicates expression of NKCC1 at E14 and P0 in vascular endothelial cells, choroid plexus endothelium, microglia, and neurons, and at P0 also in astrocytes and oligodendrocytes. Data extracted from (Loo et al. 2019). (B) Overview of the NKCC1 IR in the P1 cortex and hippocampus. (C) Strong NKCC1 IR is visible in both ramified (left panel) and ameboid (right panel) microglia. (D) Clear NKCC1 IR was found on cortical and hippocampal pyramidal neurons. (E) At P1, blood vessels were strongly immunoreactive for NKCC1, with the signal colocalizing with the pericytic marker CD13. (F) GFAP-positive cells close to ventricular wall displayed robust NKCC1 IR. LV = Lateral ventricle. Scale bars B: 100 μm, C: 5 μm, D–F: 10 μm.

To further elucidate the cellular expression patterns of NKCC1 during early development, we first analyzed NKCC1 mRNA expression in single-cell RNA-seq data from E14 and P0 mouse cortex (Loo et al. 2019). At both age points, clear expression was found in endothelial cells, choroid plexus epithelia, microglia, and neurons, and at P0 also in OLs and astrocytes (Fig. 5A). However, variation within each cell type was high, with a large fraction of cells showing no NKCC1 expression. We also tested NKCC1 mRNA expression with the ultrasensitive RNAscope in situ hybridization, which showed expression in practically all cells (Supplementary Fig. 12). However, due to the very high density of cells, a reliable quantification of expression at a single-cell level was not achievable.

Next, we visualized NKCC1 protein expression in brain sections from P1 mice. Prominent NKCC1 IR was visible across cortex and in individual cells scattered most densely around the ventricular areas (Fig. 5B). Double staining with Iba1 revealed that some of these cells are microglia (Fig. 5C), exhibiting either ramified morphology, similar to adult resting microglia, or ameboid shape, resembling the adult activated microglia.

We also detected strong, punctate NKCC1 IR that seemed to cover relatively homogenously all cortical layers and the hippocampus. Double staining with the neuronal marker MAP2 showed that part of the punctate NKCC1 IR was clearly outlining neuronal cell bodies (Fig. 5D).

Strong NKCC1 immunoreactivity was also visible on the outer borders of blood vessels, colocalizing with CD13 (Fig. 5E), which is a specific marker of brain pericytes (Crouch and Doetsch 2018). We also observed prominent NKCC1 IR close to the ventricular wall, most strongly in cells expressing GFAP (Fig. 5F). They likely represent remnants of the ventricular zone, which at this stage is involved in gliogenesis (Kriegstein and Alvarez-Buylla 2009).

### At P14, NKCC1 protein is strongly expressed in oligodendrocytes, microglia, and neural stem cells of the DG

**Fig. 6:**
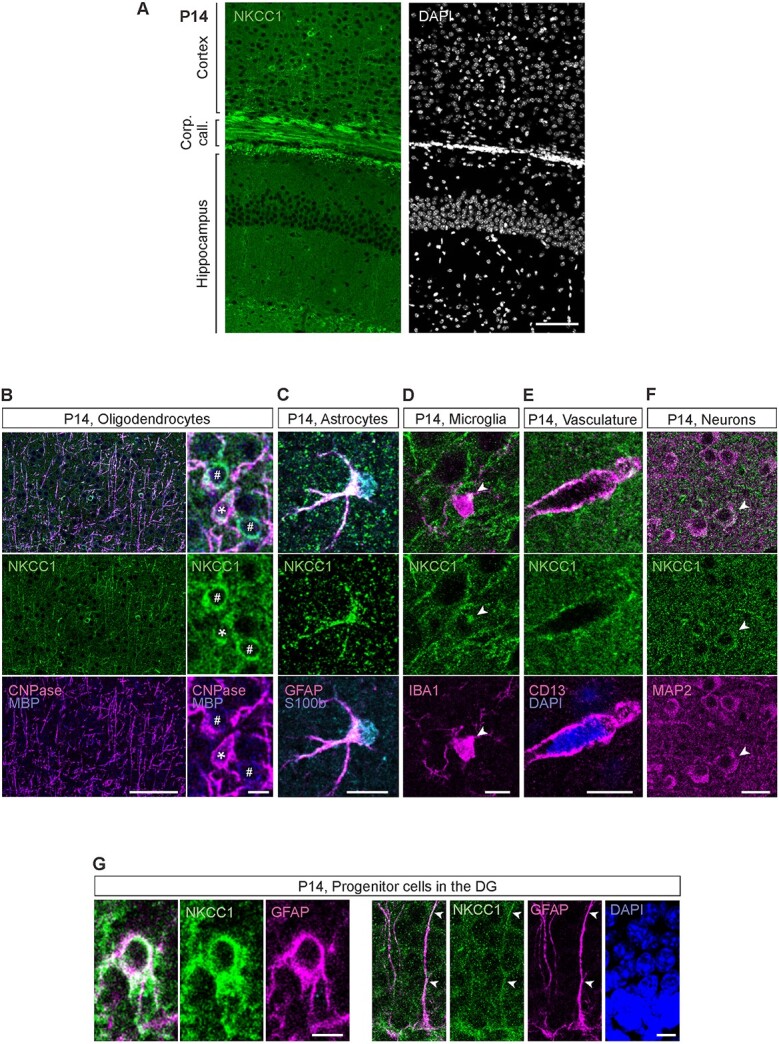
At P14, NKCC1 is strongly expressed in oligodendrocytes, microglia, vasculature, and the progenitor cells of the DG. (A) Overview of the NKCC1 IR in the P14 cortex and hippocampus. Except for the subgranular zone of the DG, majority of the NKCC1 IR colocalizes with the oligodendrocytic markers MBP and CNPase. (B) Strong NKCC1 IR in myelinated fibers and in the OL somata. *Right:* Some of the NKCC1 immunoreactive somata strongly expressed CNPase and MBP (asterisk), while others only expressed MBP (pound sign), possibly indicating different maturational stages. (C) P14 astrocytes were largely immunoreactive for NKCC1. (D) Similar to adult microglia, the P14 microglia exhibited one or a few NKCC1 IR clusters in the soma, often close to a ramification. (E) Clear NKCC1 IR was visible on blood vessels, colocalizing with the pericytic marker CD13. (F) While most cortical and hippocampal neurons did not show detectable levels of NKCC1 IR, some clearly NKCC1 positive cells (shown by the arrow head) were found in the cortex. (G) In the subgranular zone of the DG, GFAP positive neuronal progenitor cells strongly expressed NKCC1 (left). The GFAP-positive radial glia-like processes of the neuronal stem cells were also strongly NKCC1 immunoreactive (right). Scale bars A: 100 μm, B: 100 μm (left) and 10 μm (right), C, D and E: 10 μm, F: 20 μm, G: 5 μm (left) and 10 μm (right).

Compared with the perinatal stage, a different pattern emerged at P14, when the NKCC1 IR started to resemble the adult pattern, with prominent expression in myelinated fibers (Fig. 6A). We also detected very brightly stained cells within and in the proximity of the corpus callosum, and in the leading front of myelinization proceeding from the deep cortical layers toward the pial surface. Furthermore, while some of these cells were strongly positive for both CNPase and MBP, others only showed weak IR for these two markers. Nevertheless, the cells expressing low levels of CNPase and MBP showed strong IR for the pan-OL-lineage marker OLIG2 (data not shown) indicating that they represent different maturational stages of newly formed OLs (Fig. 6B).

In addition to the prominent oligodendrocytic NKCC1 expression, strong NKCC1 IR was found in the subgranular zone of the DG. Most of this signal seemed to originate from GFAP-positive cells resembling radial glia, which showed strong NKCC1 IR both in their somas and radially extending processes (Fig. 6G). These cells were negative for the marker of mature astrocytes, S100B, further suggesting that they represent radial glia-like precursor cells. However, also many mature astrocytes identified by double positivity to GFAP and S100B expressed NKCC1 strongly both perisomatically and in their processes (Fig. 6C).

Much weaker levels of NKCC1 IR were found in some, but not all, microglial cells (Fig. 6D) and in blood vessels (Fig. 6E). In most cortical and hippocampal neurons, the NKCC1 signal did not clearly stand out from the background but, in some cases, we did detect a cellular outline that corresponded to a MAP2-positive soma (Fig. 6F). In experiments with βIV-spectrin, a marker for axon initial segment (AIS), we found apparent colocalization with NKCC1 in only very few cases, and the overall data were below our preset criteria for a robust positive finding (Supplementary Fig. 13, see Discussion).

Together, the data above indicate that NKCC1 expression in specific cell types changes both qualitatively and quantitatively during postnatal development. As a whole, both neuronal and non-neuronal NKCC1 expression are detected at the perinatal stage, but only the glial expression becomes strongly upregulated during postnatal development.

## Discussion

We provide here the first properly controlled IHC analyses of cellular and developmental expression patterns of NKCC1 and its two splice variants in the mouse brain. Because of its key roles in basic cellular functions, NKCC1 is expressed in a nearly ubiquitous manner outside the brain (Russell 2000; Delpire and Gagnon 2018), and the RNA-seq data suggest similarly extensive expression patterns of NKCC1 protein in the brain (see Fig. 2). However, we were still astonished by the amazingly wide-ranging NKCC1-IR in the brain detected in the present study. Clearly, much of the overall NKCC1-IR signal is generated in the thin processes of numerous cell types which are tightly intermingled and superimposed, underscoring the fundamental status of NKCC1 in (sub)cellular physiology. However, this also makes the IHC analysis of its cellular and subcellular localization particularly challenging in native brain tissue. The widespread expression patterns will also by necessity create a more or less homogenous background even in thin sections, which obviously leads to a compromised S/N ratio in NKCC1-IR analyses of distinct cellular and subcellular structures. Therefore, we have reported and discussed our data in a manner in which colocalization with other molecules was inferred in an extremely careful (i.e. “conservative”) manner, thus preventing false positive conclusions, which we consider counterproductive at this early stage of IHC research on NKCC1 in the brain parenchyma. In future work, higher resolution methods such as superresolution imaging or electron microscopy will be needed for definite visualization of NKCC1 in subcellular structures.

The present data were obtained using a KO-controlled approach (Fritschy 2008) with carefully validated custom-made antibodies and a procedure including robust antigen retrieval. These data were further corroborated by examining cellular NKCC1 mRNA profiles. In the perinatal forebrain, NKCC1 protein is expressed at high levels in neurons, microglia, and glial progenitor cells as well as in the vasculature, while in the adult, OLs, microglia, and astrocytes show salient NKCC1 protein expression. Data based on the use of an NKCC1a-selective antibody and parallel mRNA analyses showed that NKCC1a is the main splice variant in glial cells, while NKCC1b is predominant in neurons. A further major finding was that NKCC1 protein is indeed upregulated during brain development and, in line with previous in situ data (Hübner et al. 2001), this upregulation is attributable to the glial splice variant NKCC1a. Taken together, these observations give *a firm and unequivocally negative answer* to the long-standing question whether tissue-level analyses of total NKCC1 protein (and/or mRNA) expression levels provide any meaningful information on neuronal NKCC1 expression during brain development and disease (see Introduction). Below, we will discuss the NKCC1 expression patterns in distinct cell types in the brain parenchyma and vasculature.

### Neurons

At a very early postnatal stage (P1), our immunostainings showed a strong NKCC1 signal in neuronal somata, especially in the neocortex. A weaker somatic localization was still detectable in some neocortical neurons at P14, but not in the adult. This is in line with functional studies, which show a gradual disappearance of bumetanide-sensitive chloride transport and consequent depolarizing GABA responses during the maturation of cortical and hippocampal neurons both in vivo and in vitro (Yamada et al. 2004; Kaila, Price, et al. 2014; Sulis Sato et al. 2017).

There is a lot of functional evidence on the presence of NKCC1 in different axonal compartments of numerous types of neurons in adolescent and adult animals. In the AIS of pyramidal neurons, GABA induces depolarization at P11–P39 (Szabadics et al. 2006; Khirug et al. 2008), an effect that disappears at the periadolescent stage (by P40 in the mouse somatosensory cortex) (Rinetti-Vargas et al. 2017; Pan-Vazquez et al. 2020). In double stainings with the AIS marker βIV-spectrin in P14 animals, we sporadically detected NKCC1 IR at the AIS of cortical neurons that was of slightly higher intensity than in the surrounding tissue, implying a possible colocalization. Furthermore, GABA_A_Rs can mediate the depolarization of presynaptic glutamatergic terminals on hippocampal CA3 neurons (Jang et al. 2006), in the hypothalamus (Jang et al. 2001) and in parallel fibers in the cerebellum (Stell et al. 2007). We could not detect any clear colocalization with the NKCC1 IR in double stainings with the glutamatergic presynaptic markers VGLUT1 and VGLUT2 in adult animals (Supplementary Fig. 7A and B). Our data might suggest the colocalization of NKCC1 with the presynaptic GABAergic marker VGAT (Supplementary Fig. 7C), but the evidence is not strong enough for a solid positive conclusion. It has been known for a long time that NKCC1-mediated Cl^−^ loading takes place in the axon proper, e.g. in squid axons (Russell 1983) and in the mouse unmyelinated sural nerve (Bonalume et al. 2021). Neuronal NKCC1 is also required for maintaining the integrity of myelinated axons in the zebrafish PNS (Marshall-Phelps et al. 2020). However, detecting NKCC1 in thin unmyelinated axons (and myelinated axons; see below) using IHC was not possible in the present study.

While we could not definitely show neuronal NKCC1 IR in adults, the mRNA was clearly visible in most neurons when using ultrasensitive in situ hybridization, and our qPCR results indicate that the expression levels of the neuronal splice variant NKCC1b remain relatively stable during postnatal development. A possible explanation for these observations is that mature neurons produce and retain NKCC1 mRNA under normal conditions and that significant protein translation in the somato-dendritic compartment, as evidenced by a large number of studies on depolarizing GABA actions, would only start under pathophysiological conditions, such as seizures or stroke (Kaila, Price*,* et al. 2014, and see Introduction). Indeed, rapidly expanding evidence indicates that RNA binding proteins can tightly regulate protein expression in specific subcellular compartments (see Schieweck et al. 2021). Here, it should be noted that distinct members of the CCC family are differentially trafficked into subcellular domains in healthy adult pyramidal neurons. NKCC1 is located within the axonal compartment as observed in functional studies, while KCC2 is trafficked to somatodendritic sites including dendritic spines (Báldi et al. 2010; Fiumelli et al. 2013; Virtanen et al. 2021). Work on epileptic tissue shows that these trafficking patterns of CCCs are likely to change following neuronal trauma (Huberfeld et al. 2007).

The present data show that *NKCC1b is the main neuronal splice variant*. The preferential expression of NKCC1b mRNA splice variant in neuronal tissues has been reported before in mice (Randall et al. 1997; Gregoriades et al. 2019) and humans (Vibat et al. 2001; Morita et al. 2014). Notably, our qPCR, WB, and IHC data all together point to non-neuronal (i.e. mainly glial) NKCC1a as the major splice variant in the adult cortex. Interestingly, and despite the fact that expression of NKCC1 tends to be enhanced in various kinds of cell cultures (Russell 2000), the pure glial cultures examined here expressed NKCC1a mRNA only, while mixed cultures with neurons and glia had both splice variants. These data point to a powerful control of cell-type-specific expression of NKCC1a and NKCC1b, which is retained even during proliferation and growth in vitro.

### Oligodendrocytes

Both our immunostainings and RNA data indicate that NKCC1 is highly expressed in developing and mature OLs of the adult mouse brain. We observed a particularly strong NKCC1 signal in the OLs at P14, when the myelination rate is at its peak (Hamano et al. 1998; Spitzer et al. 2019). In the adolescent mouse hippocampus, NKCC1 activity in OLs has been reported to mediate changes in axonal conduction, which in turn modifies synaptic plasticity (Yamazaki et al. 2021). Notably, we observe strong NKCC1 IR also in the myelinated fibers of mice older than P90. OLs are critical for normal function of myelinated axons by buffering extracellular potassium juxta-axonally via Kir4.1-mediated removal (Larson et al. 2018; Schirmer et al. 2018), which in combination with our results indicate dynamic ion transport at the neuron-OL interface. Moreover, NKCC1 is responsible for depolarizing GABA responses evoked by GABAergic interneurons in OLs (Wang et al. 2003). However, we did not detect NKCC1 in OPCs in colocalization experiments with the OPC marker PDGFRα, although NKCC1 has been shown to regulate the proliferation and maturation of OPCs in neonatal mice (Zonouzi et al. 2015). It is obvious that functional assays (mainly based on the potent NKCC1 blocker, bumetanide, applied in vitro or directly to brain tissue; see Löscher and Kaila 2022) have a higher sensitivity than IHC in detecting the mere presence of NKCC1 in various cell types.

Most of the NKCC1 IR in the adult mice colocalized with myelin markers, indicating that OLs express a high level of NKCC1 protein, in agreement with their high expression of the mRNA. Given the small diameter of the ensheathed axons, their potential contribution (see section on neurons above) to the total NKCC1 IR is likely to be very small. An important question for future work on OL ultrastructure is the subcellular distribution of NKCC1, especially the spatial relationships between NKCC1 and GABA_A_Rs in the complex morphology of these cells.

We were a bit surprised to find that while the IR with panNKCC1 antibodies (GpA and RbC) indicated a high total level of NKCC1 in OL cell bodies, the NKCC1a-specific IR was very weak. A simple explanation to account for this is that the NKCC1b mRNA seen in the RNA-seq analysis in OLs (about 29% of total NKCC1 in OLs) is translated to protein, which is retained as the only splice variant in the soma. Indeed, as described above in the context of neurons, subcellular heterogeneity is a common property of members of the CCC family. Another possibility is that the exon-21 encoded region could be specifically masked in intracellular compartments, for instance by posttranslational modifications. Recent work has shown that oligodendrocytes are of paramount importance in brain functions (De Faria et al. 2021; Yalçın and Monje 2021; Munyeshyaka and Fields 2022). We consider it highly likely that future work on these cells will lead to important findings on the roles of oligodendrocytic NKCC1 in CNS development, plasticity, and disease (see also Castelijns et al. 2020).

### Microglia

In individual adult microglial cells, we consistently observed one or a few somatic or proximally located clusters of NKCC1 IR, with an overall appearance that is not expected from a transmembrane protein. It is likely that these clusters are intracellular and may represent a reserve pool, ready to be transported to specific subcellular locations upon microglial activation, which is a very fast process [down to 8 min for phagosome formation (Mazaheri et al. 2014)]. This would be similar to trafficking of cytoplasmic KCC2 (in the absence of protein synthesis) to the plasma membrane in neonatal neurons, triggered by a single seizure episode (Khirug et al. 2010). Indeed, microglial NKCC1 has been shown to both regulate the transformation of microglia to a reactive state and activate the lesion-induced recruitment of their processes (Tóth et al. 2022). NKCC1 is involved in shaping cellular movement and morphology in numerous kinds of cells (Russell 2000), for instance in the migration of glioblastoma, in which it localizes in the distal edge of their extending processes (Garzon-Muvdi et al. 2012).

At P1, strong NKCC1 IR covered the soma and ramifications of microglial cells. In the proximity of ventricular areas, many of these cells had an ameboid morphology, resembling adult activated microglia (Kettenmann et al. 2011; Izquierdo et al. 2019). In the neonatal mouse and rat subventricular zone, activated microglia regulate postnatal neurogenesis and myelination (Shigemoto-Mogami et al. 2014; Wlodarczyk et al. 2017; Santos and Fields 2021). Thus, in the immature brain, microglial NKCC1 might control numerous developmental mechanisms (Michell-Robinson et al. 2015).

### Astrocytes

In adult astrocytes, NKCC1 IR was mainly found in the proximal ramifications. NKCC1 has been shown to regulate the LTP-associated withdrawal of perisynaptic astrocytic processes together with the actin regulatory protein cofilin (Henneberger et al. 2020). Notably, P14 astrocytes showed a qualitatively similar but more intense NKCC1 IR than adults, which may reflect the massive functional reorganization of cortical and hippocampal structures around this postnatal day (see, e.g. Erecinska et al. 2004; Ruusuvuori et al. 2013).

### Progenitor cells in the DG

Strong NKCC1 IR was detected in the developing DG. Most of this signal seemed to originate from radial glia-like precursor cells, identified by their radial morphology and expression of GFAP but not of the astrocytic marker S100B (Raponi et al. 2007). Uptake of Cl^−^ by NKCC1 has been shown to regulate proliferation and cell cycle progression of neuronal progenitors during early development (LoTurco et al. 1995; Haydar et al. 2000; Sun et al. 2012; Magalhães and Rivera 2016). We also saw strong NKCC1 signal at P1 in the GFAP-positive cells lining the ventricular wall, likely representing remnants of the ventricular zone, which at this stage is involved in gliogenesis (Tramontin 2003; Kriegstein and Alvarez-Buylla 2009). Indeed, NKCC1 has been implicated in the proliferation of numerous kinds of cells, including OPCs (Russell 2000; Zonouzi et al. 2015; Yu et al. 2018).

### Vasculature and pericytes

The strong vascular NKCC1 signal that we observe in the immature brain is interesting because the mouse brain microvascular network undergoes extensive expansion and refinement during the first postnatal month, with vessel branching and proliferation of pericytes and endothelial cells (Erecinska et al. 2004; Harb et al. 2013). The vascular NKCC1 IR in the perinatal brain seems to colocalize with the pericyte marker CD13, but at least part of the NKCC1 IR may originate from other nearby cells, such as those in the endothelium. Notably, NKCC1 is in a key position in the regulation of contractility of pericytes (Korte et al. 2022) and vascular smooth muscle cells (Jiang et al. 2004).

Taken together, our results show for the first time cellular patterns of NKCC1 protein expression in the developing and mature mouse forebrain, providing a much-needed structural framework for NKCC1 research. Strikingly high IR is seen in numerous cell types, particularly in the OL lineage which, accordingly, are known to generate depolarizing GABA responses (Berger et al. 1992; Káradóttir and Attwell 2007). Some studies, based on NKCC1 antibodies and procedures which have not been KO controlled, have shown apparently high levels of NKCC1 specifically in neuronal somata in healthy adult tissue with no obvious IR in other cell types (see, e.g. references in Virtanen et al. 2020). However, the present work demonstrates the opposite: NKCC1 IR is absent or extremely low in the somatic compartments of healthy adult neurons, which is consistent with a large number of studies on bumetanide actions on E_GABA_ (Blaesse et al. 2009; Kaila, Price*,* et al. 2014). Moreover, the strong NKCC1 expression in non-neuronal cells means that measuring NKCC1 mRNA and/or protein levels in homogenized brain tissue samples (which obviously contain numerous cell types) cannot be used in attempts to estimate developmental or disease-associated changes in neuronal NKCC1 expression levels or “NKCC1/KCC2 ratios” (Virtanen et al. 2020).

The cell-type-specific expression patterns of NKCC1a and NKCC1b, which turned to be the main glial and neuronal splice variants, respectively, will provide crucial molecular-level information for future work designed to examine changes in NKCC1 expression patterns in CNS development, plasticity, and disease. Obviously, this is highly relevant also when re-evaluating published data on genetic manipulation of neuronal NKCC1, which has in some previous studies been done by nonspecific knock-down methods that must have unintentionally targeted NKCC1 in both neuronal and glial cells (cf. Virtanen et al. 2020 and; Löscher and Kaila 2022 for further details and references).

Finally, our results have obvious pharmacological implications on work aiming at the development of drugs that would block NKCC1 in central neurons (for review, see Löscher and Kaila 2022). Even if bumetanide or its derivatives would reach the brain parenchyma under in vivo conditions, they would inhibit NKCC1 functions in practically all kinds of cells within the brain parenchyma. This grave problem (see also Puskarjov et al. 2014) has not been recognized in most of the past and even recent experimental work. Theoretically, a drug that is selective for the NKCC1b splice variant would mainly target neuronal NKCC1. This might be a very difficult aim to achieve, given the lack of success in developing molecules which would be more selective for NKCC1 than for the kidney-located isoform, NKCC2 (Löscher and Kaila 2022). However, the very recent breakthroughs in de novo design of protein-binding proteins provide much hope for the next generation of both neuron-selective and neuron-sparing NKCC1-blockers in the foreseeable future (Jumper et al. 2021; Cao et al. 2022).

## Supplementary Material

Kurki_Supplement_Final_bhac470
