# Supplementary material for: Expression patterns of NKCC1 in neurons and non-neuronal cells during cortico-hippocampal development
Source: Cereb Cortex. 2022 Dec 27;33(10):5906–23. doi: 10.1093/cercor/bhac470 (PMC10183754; doi:10.1093/cercor/bhac470)
Supplement: Kurki_Supplement_Final_bhac470 [file kurki_supplement_final_bhac470.zip › Kurki_Supplement_Final_bhac470.docx]

**Supplementary Information**

**Expression patterns of NKCC1 in neurons and non-neuronal cells during cortico-hippocampal development**

Samu N Kurki^1,2^, Pavel Uvarov^1,2,^, Alexey S Pospelov^1,2^, Kalevi Trontti^2,3,4^, Antje K Hübner^5^, Rakenduvadhana Srinivasan^1,2^, Masahiko Watanabe^6^, Iiris Hovatta^2,3,4^, Christian A Hübner^5^, Kai Kaila^1,2^, Mari A Virtanen^1,2^

**Corresponding author:**

Prof. Kai Kaila

Faculty of Biological and Environmental Sciences

Molecular and Integrative Biosciences, and Neuroscience Center (HiLIFE)

PO Box 64, 00014 University of Helsinki, Finland

Email: [kai.kaila@helsinki.fi](mailto:kai.kaila@helsinki.fi), phone: +358 40 725 6759

**
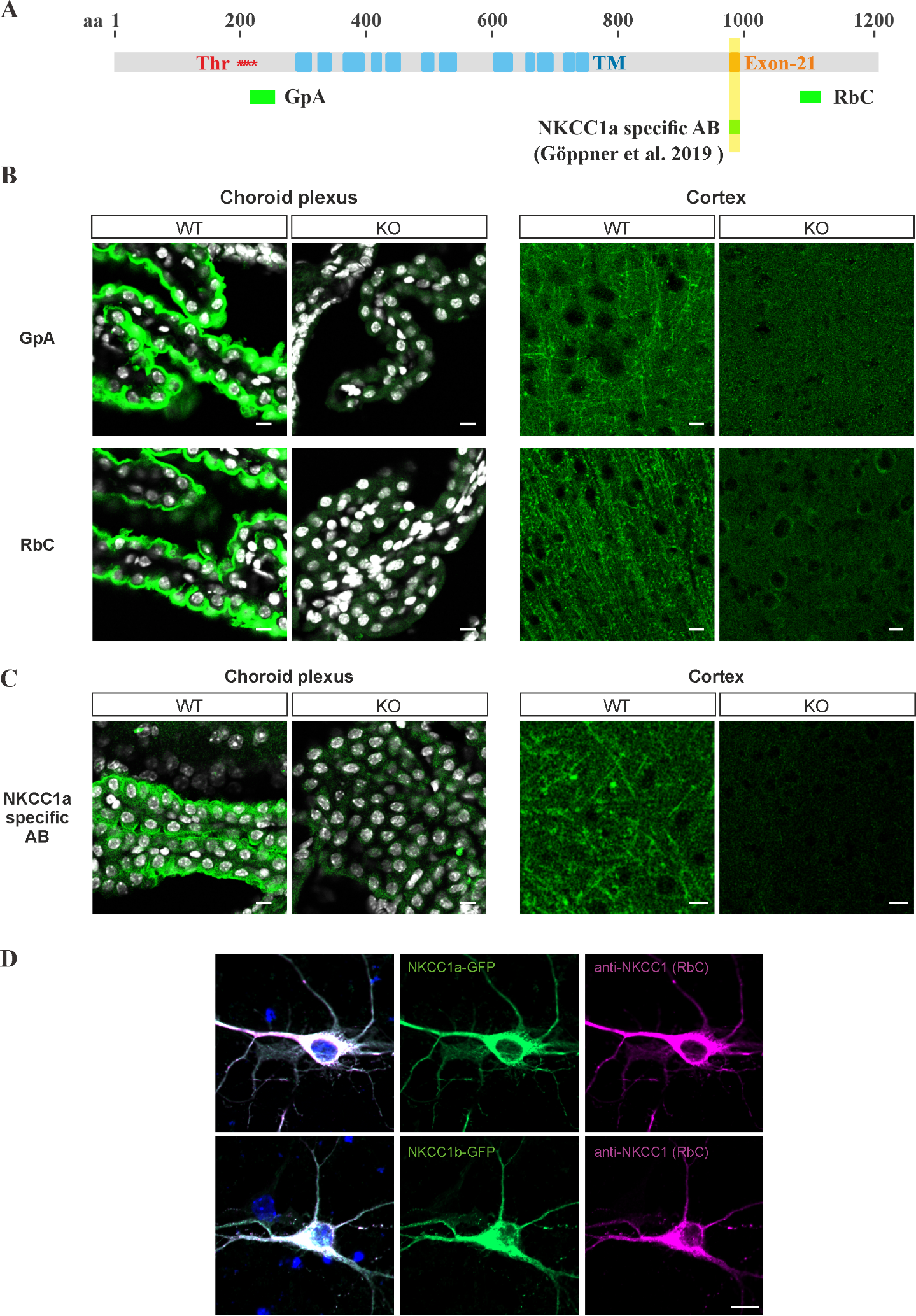
Supplementary Figures**

**Supplementary Figure 1: NKCC1 antibodies targeting different regions of the protein show specific knock-out controlled IR**

**A:** NKCC1 regions targeted by the different antibodies. GpA: affinity-purified polyclonal guinea pig antibody against aa 237-275 of the mouse NKCC1. RbC: affinity-purified polyclonal rabbit antibody against aa 1108-1141 of the mouse NKCC1. NKCC1a specific antibody (Göppner et al., 2019): affinity-purified rabbit polyclonal antibody against aa 977–991 of the mouse NKCC1. Predicted transmembrane domains are shown in blue, and the exon-21 encoded region is highlighted in orange. Asterisks indicate the five well-studied phosphorylation sites (Thr197, Thr201, Thr206, Thr211, and Thr224). Amino acid numbering corresponds to the mouse NKCC1. **B:** Both GpA and RbC produced highly similar results. Strong specific signal was visible in the apical side of the choroid plexus epithelium. In parenchymal regions, myelinated fibers were brightly stained, and small glial cell bodies were visible. In contrast, the non-specific background pattern in the KO-tissue was weak and homogenous. **C:** The NKCC1a specific antibody also gave a strong signal in the choroid plexus and brain parenchyma of the WT animals, but not in the KOs. **D:** PanNKCC1 antibody RbC recognizes both NKCC1 splicing variants in cultured cells overexpressing either NKCC1a or NKCC1b. *Scale bars 10 μm*

**
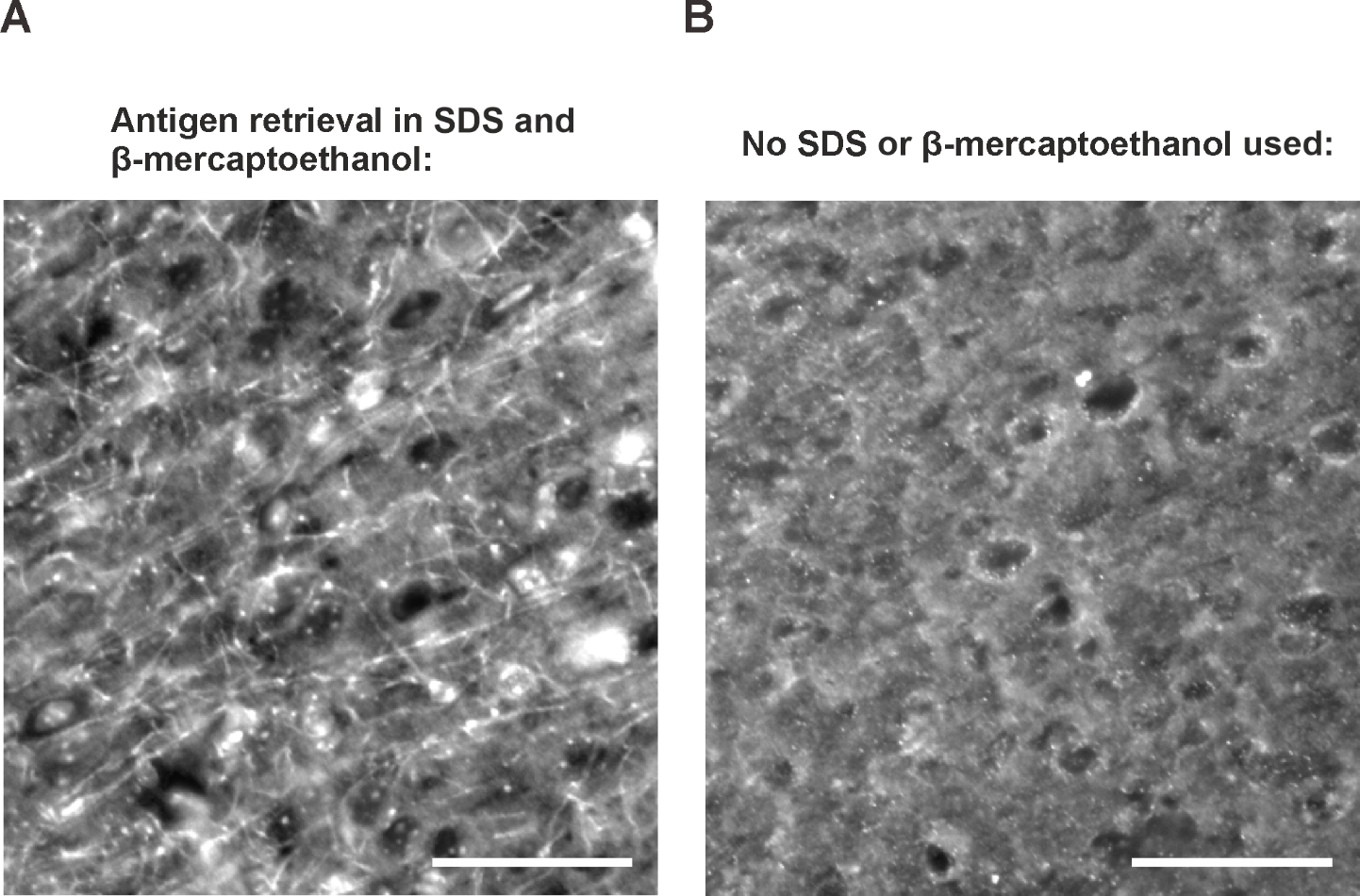
**

**Supplementary Figure 2: NKCC1 IR only appears after strong antigen retrieval**

**A**: NKCC1 staining with the RbC antibody in adult mouse cortical sections after antigen retrieval in SDS and β-mercaptoethanol (5 min incubation in 1 % SDS and 8 % β-mercaptoethanol). **B**: No clear NKCC1 IR was seen in sections when the SDS / β-mercaptoethanol antigen retrieval step was omitted. *Scale bars 50 μm*

**
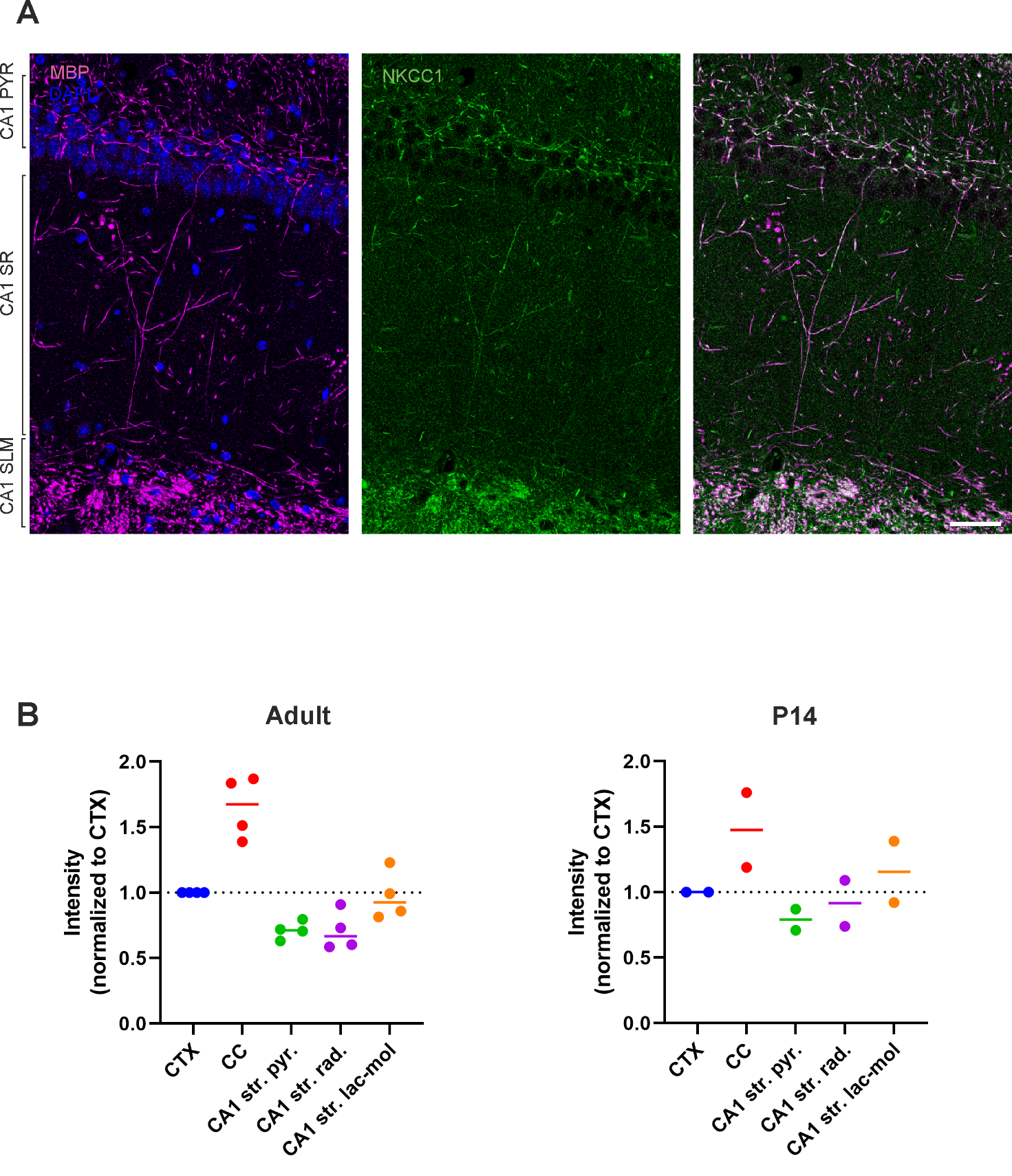
**

**Supplementary Figure 3: Relative NKCC1 IR intensity in cortico-hippocampal system and examples of the hippocampal NKCC1 expression patterns**

**A:** Strong hippocampal NKCC1 IR in *CA1* *stratum lacunosum-moleculare* largely colocalizes with the myelin marker MBP. Weaker and scattered NKCC1 IR *stratum pyramidale and stratum radiatum.* **B**: Quantification of NKCC1 IR intensity normalized to cortical values in adult and P14 animals. *Scale bar 50 μm*

**
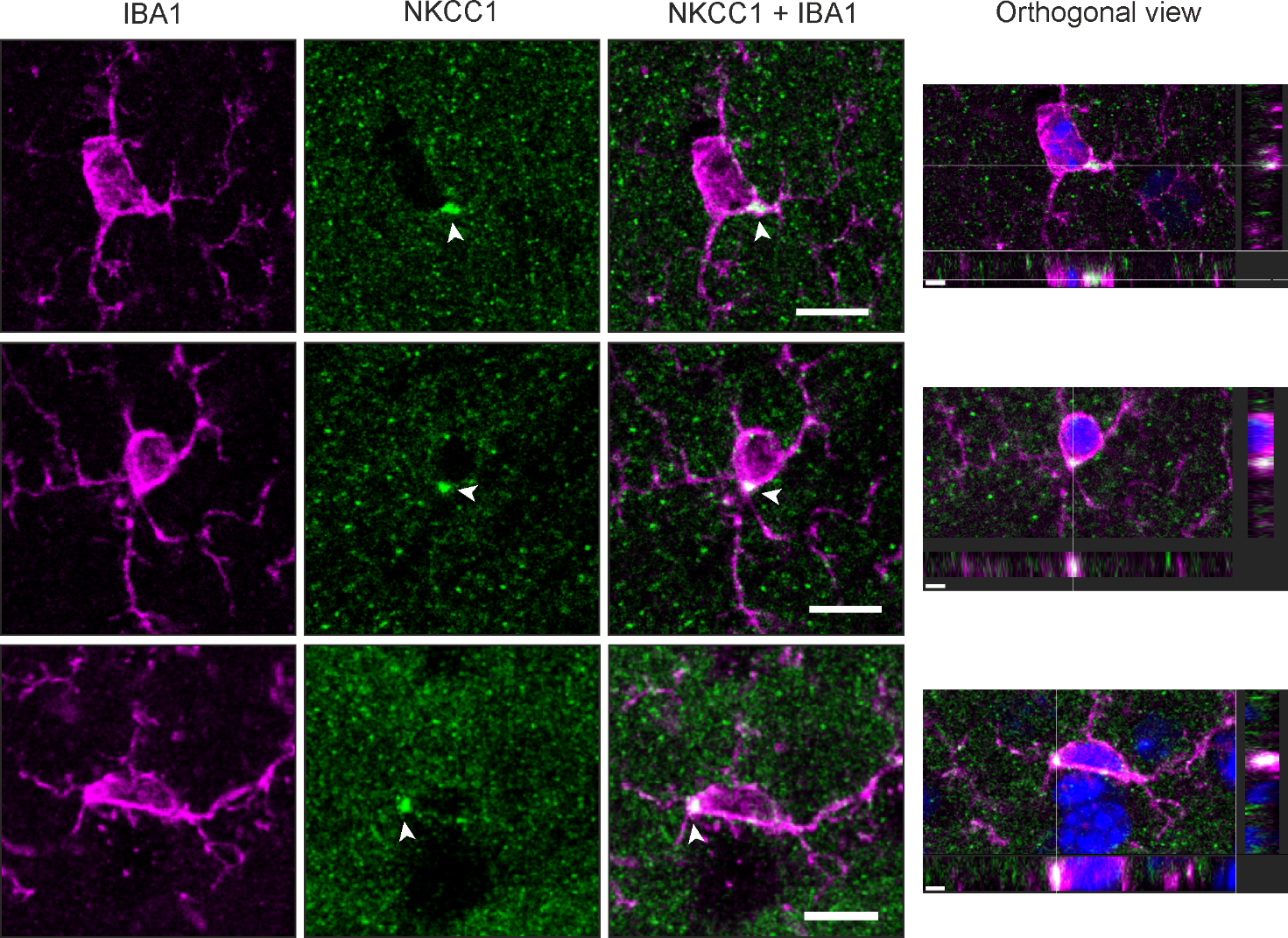
**

**Supplementary Figure 4: Extended data on microglial NKCC1 IR in the adult mouse**

NKCC1 IR was consistently found in microglial cells, detected by their Iba1 expression. Typically, one or few NKCC1 IR clusters were seen in the soma, often close to a ramification (arrowhead). Orthogonal view shows NKCC1 and IBA1 IR simultaneously in XY, XZ and YZ planes. *Scale bars 10 μm; 3 μm in the orthogonal views*

**
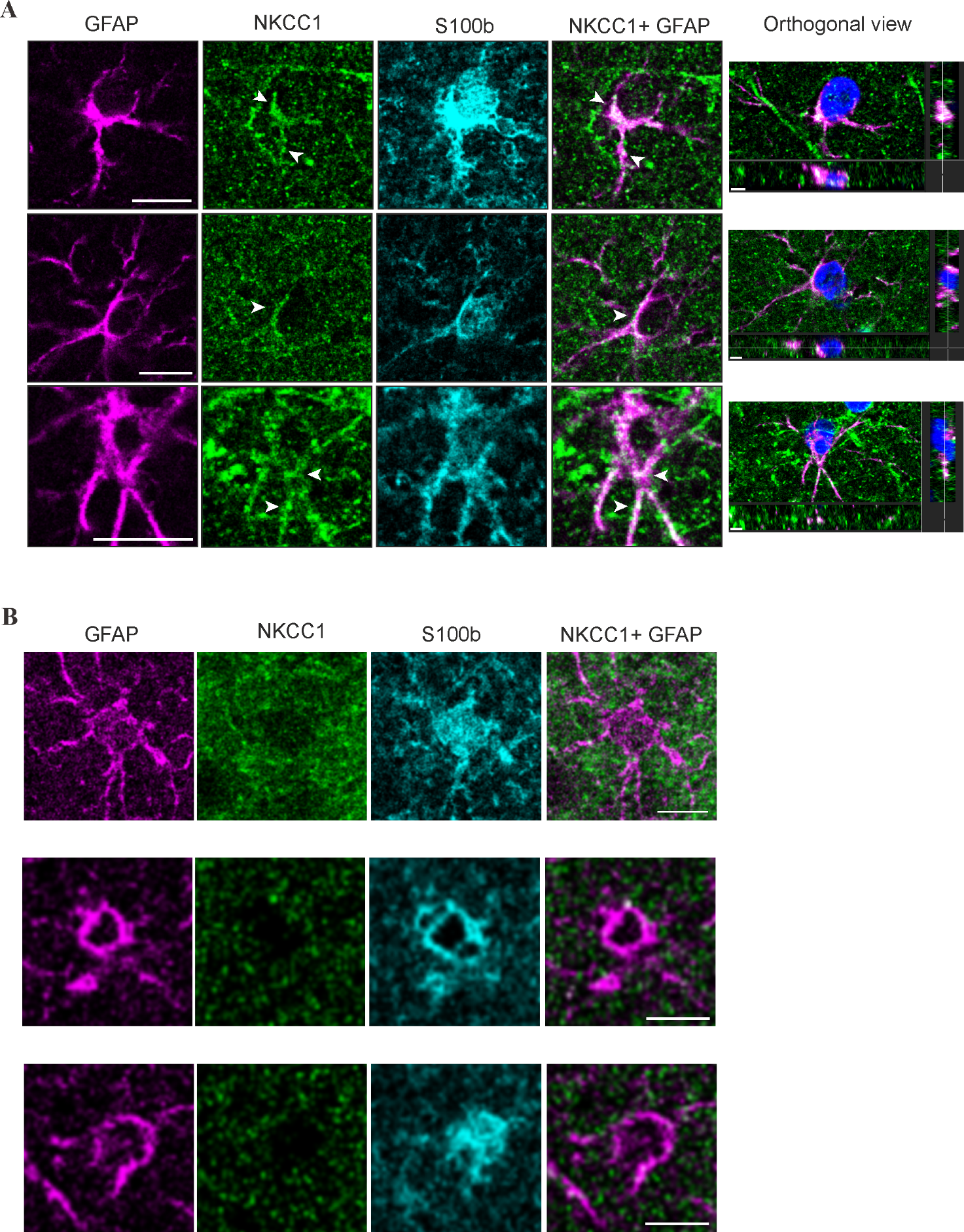
**

**Supplementary Figure 5: Extended data on astrocytic NKCC1 IR in the adult mouse**

**A:** Astrocytes, identified by expression of both GFAP and S100B, consistently showed NKCC1 expression in some of their rami and perisomatically (arrowheads). Orthogonal view shows NKCC1 and GFAP IR simultaneously in XY, XZ and YZ planes.

**B:** Around 15% of astrocytes did not show reliably detectable NKCC1 IR. *Scale bars A: 10 μm and 3 μm in the orthogonal views, B: 10 μm*

**
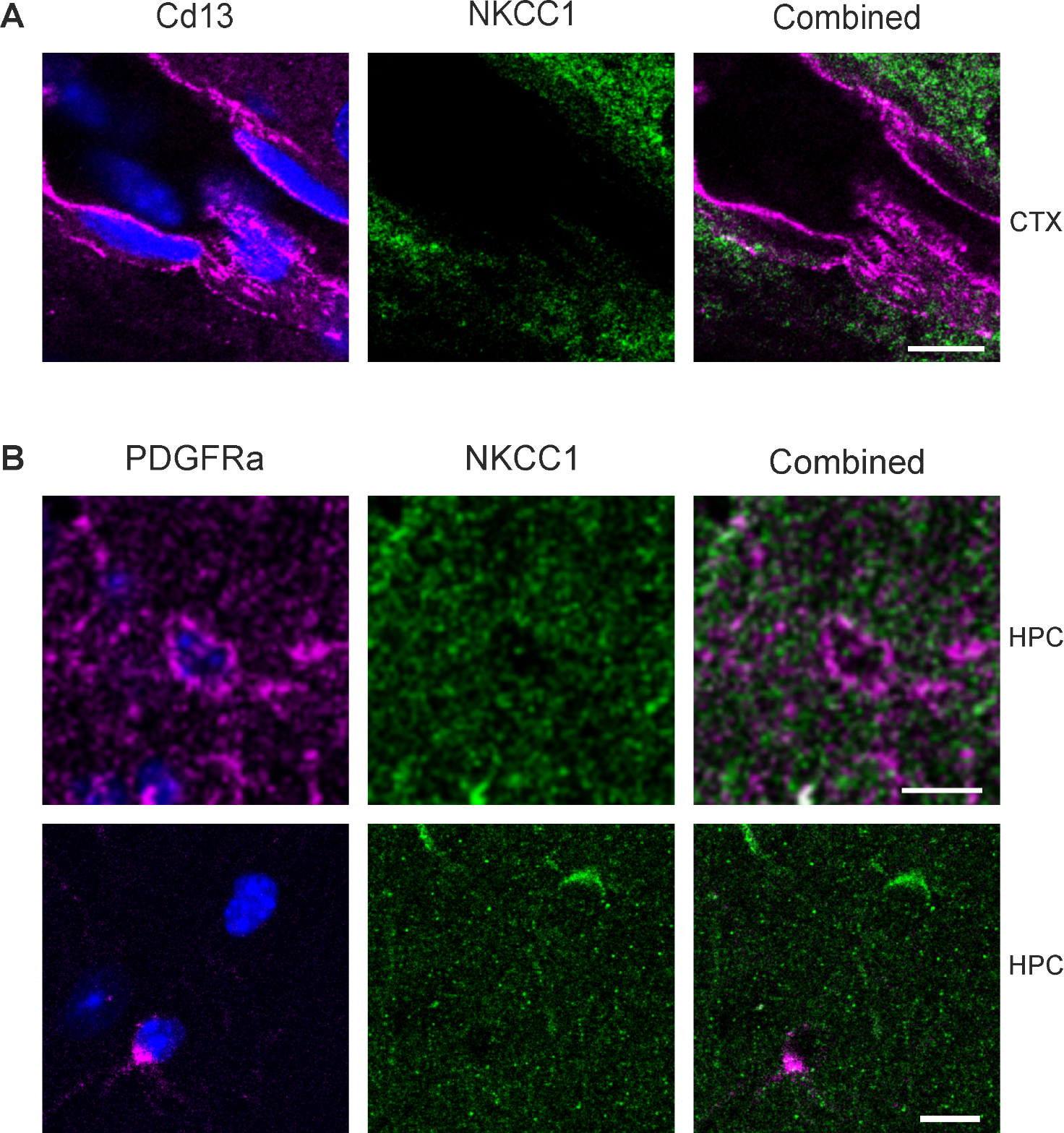
**

**Supplementary Figure 6: NKCC1 IR could not be detected in adult pericytes or OPCs**

NKCC1 IR in **(A)** pericytes detected by marker CD13 and **(B)** OPCs identified by marker PDGFRα. *Scale bars 10 μm*

**
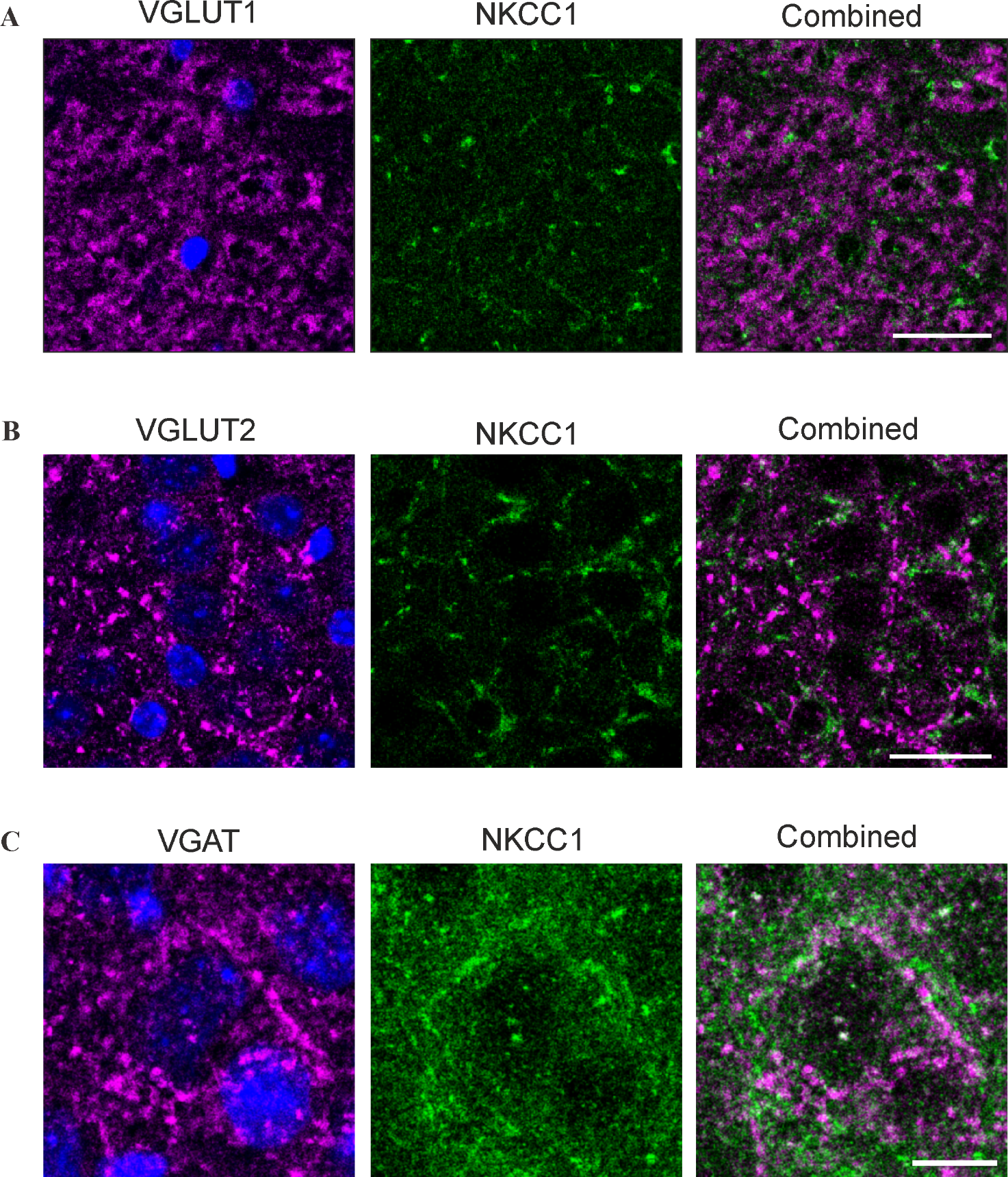
**

**Supplementary Figure 7: NKCC1 IR could not be detected in glutamatergic presynaptic terminals identified by VGLUT1 and VGLUT2 markers, whereas it was ambiguous in GABAergic presynaptic terminals identified by VGAT**

NKCC1 IR in presynaptic glutamatergic terminals identified by **(A)** VGLUT1 and **(B)** VGLUT2 in hippocampus, and GABAergic terminals identified by **(C)** VGAT in cortex. *Scale bars A, B: 20 μm; C: 10 μm*

**
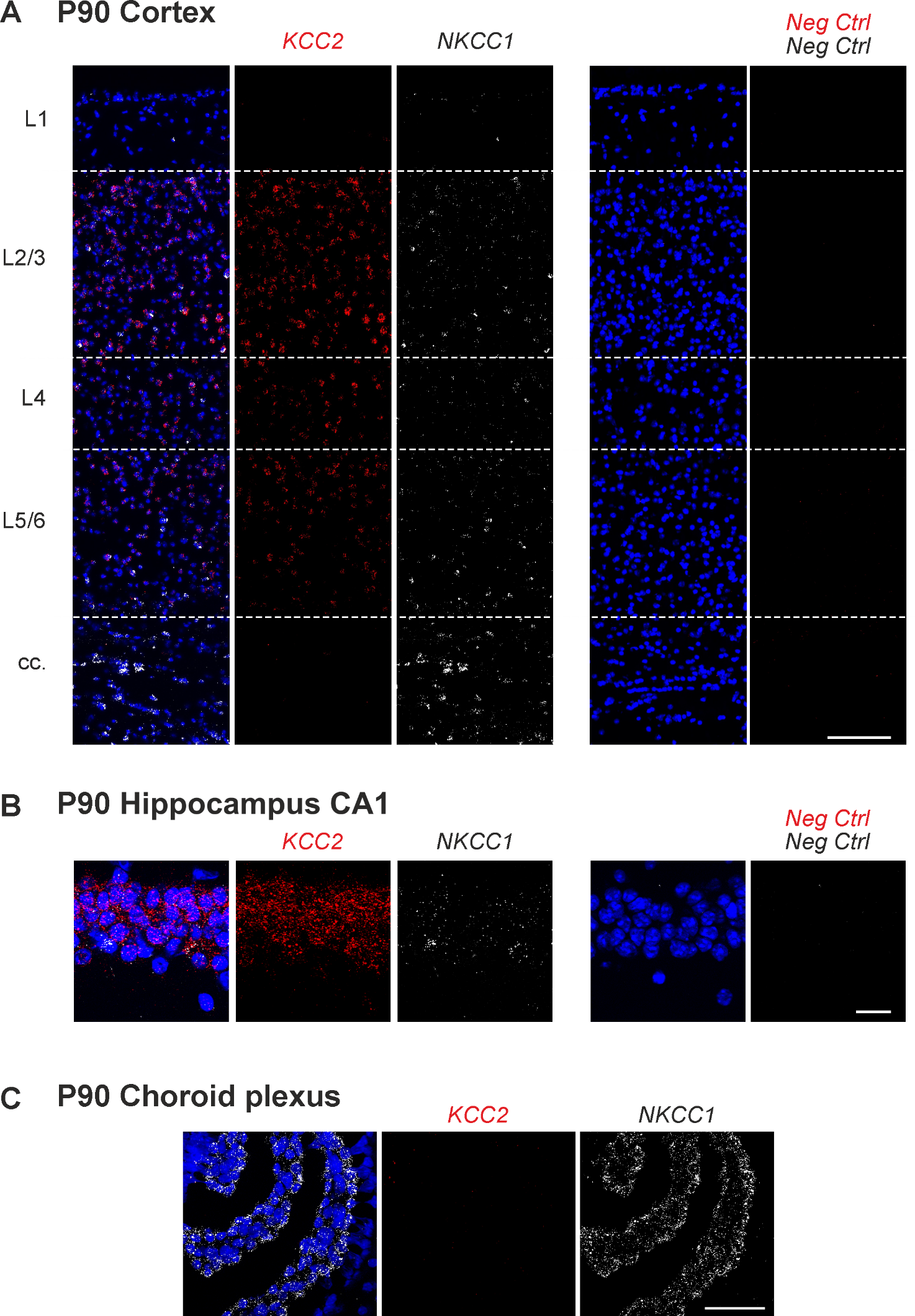
**

**Supplementary Figure 8: Extended data on NKCC1 mRNA expression in the P90 somatosensory cortex, hippocampus and choroid plexus shown by the ultrasensitive RNAscope *in situ* hybridization**

**A:** NKCC1 mRNA expression in neuronal (KCC2 positive) and non-neuronal (KCC2 negative) cells in the somatosensory cortex. Individual non-neuronal cells with very high NKCC1 expression are scattered across cortical layers, and most prominently in the corpus callosum. A much weaker NKCC1 mRNA signal can be seen in most neurons. Negative control probes show no detectable signal in either channel. **B:** Weak NKCC1 mRNA signal was seen in hippocampal CA1. **C:** The strong NKCC1 mRNA signal in the choroid plexus provides a positive control for the RNAscope procedure. *Scale bars A: 100 μm, B: 20 μm, C: 50 μm*

**
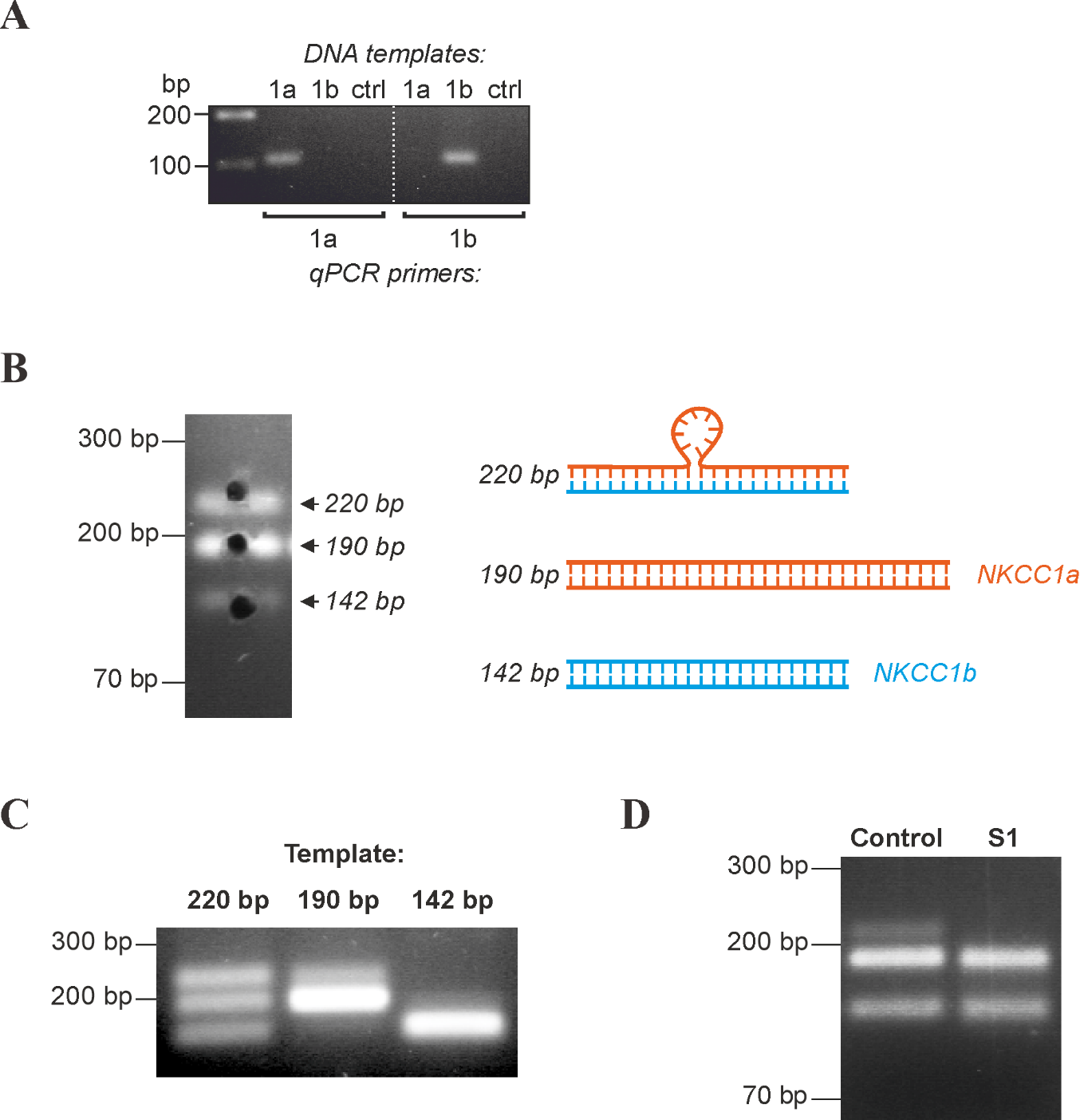
**

**Supplementary Figure 9. Specificity of the PCR primers**

**A:** Specificity of the splice variant specific primers was tested using NKCC1a and NKCC1b encoding templates. As expected, NKCC1a specific primers only detected NKCC1a, whereas NKCC1b primers specifically amplified the NKCC1b product. **B:** PCR amplification with the common primers produces three bands (220 bp, 190 bp, and 142 bp). Each band was isolated for further analysis (holes show the location of pipet punctures). Schematic drawing of the proposed identity of the bands. The 220 bp band is expected to consist of NKCC1a/NKCC1b heterodimers. **C:** In the first experiment, each band isolated from the gel shown in panel B was further amplified with the common primers. The 190 bp template and 142 bp template generated products corresponding to their original size. In contrast, the 220 bp template produced equally strong bands of all three sizes, showing that the 220 bp band contains both NKCC1a and NKCC1b products as non-specific heterodimers. The weaker band in 190 bp template is most probably either single stranded DNA, or weak contamination from the 142 bp band. **D:** In the second experiment, we amplified the 220 bp band with common primers, and then used S1 Nuclease (20 U/reaction) to degrade single stranded DNA. The third band fully disappeared, confirming the looped structure of the product.

**
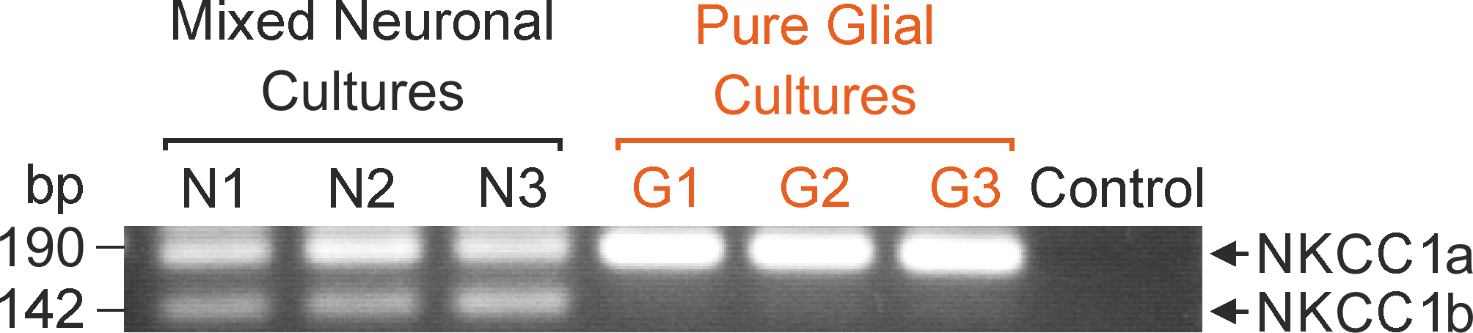
**

**Supplementary Figure 10: Both NKCC1a and NKCC1b mRNA are detected in mixed neuronal-glial cultures, whereas NKCC1b is absent in pure glial cultures**

NKCC1a is expressed in both mixed neuronal cultures and in pure glial cultures, whereas NKCC1b is only detected in mixed neuronal cultures. Each column represents a culture dish. The experiment was repeated with four independent cultures, and the results were identical.

**
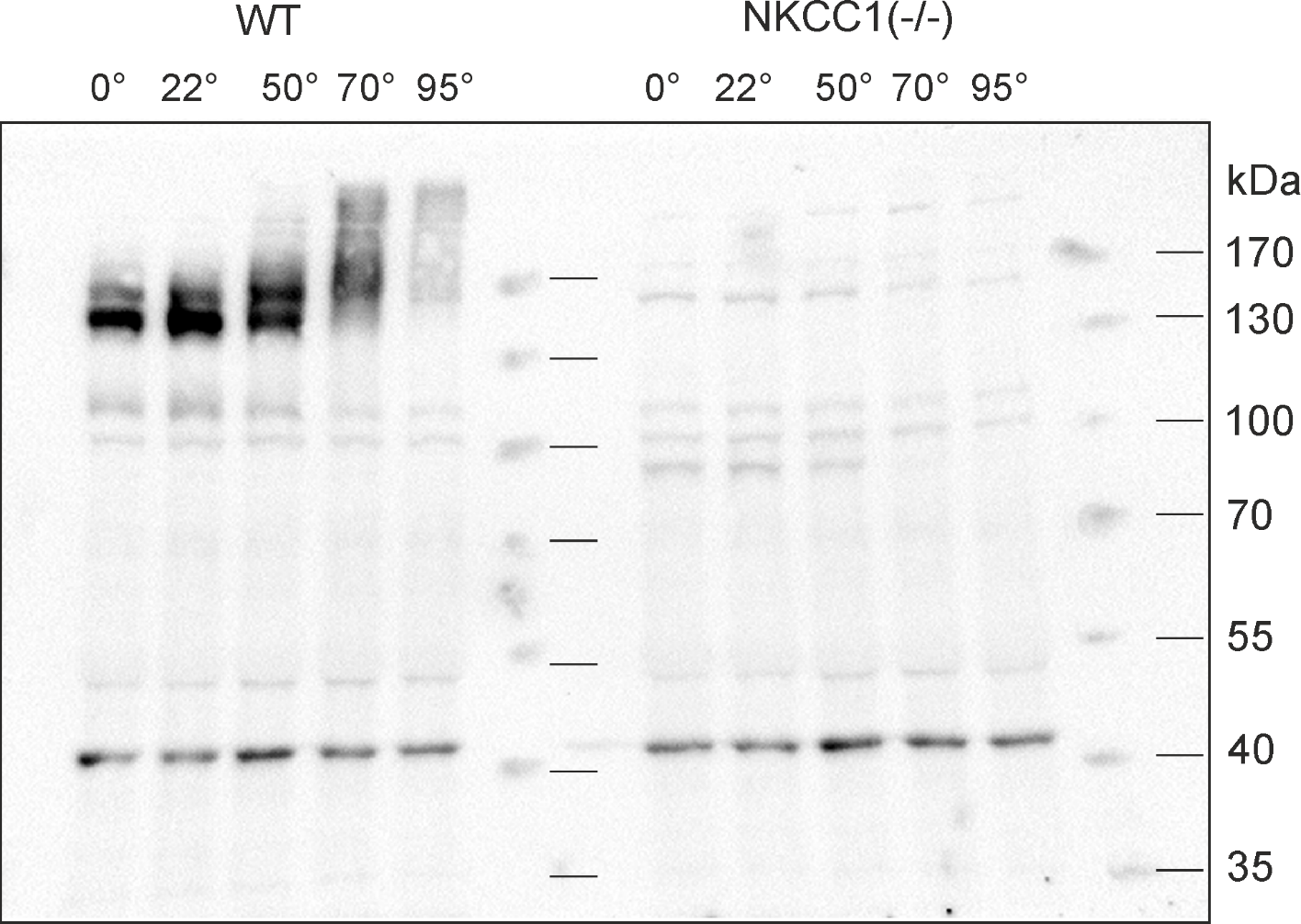
**

**Supplementary Figure 11: Validation and optimization of NKCC1 Western blot protocol**

The RbC NKCC1 antibody specifically detects endogenous NKCC1 protein in wild type but not in NKCC1^-/-^ cortical lysates. Even a brief (5 min) heating of the protein lysates before loading them on the SDS-PAGE at temperatures higher than 50 °C, resulted in a pronounced NKCC1 aggregation and consequent masking of the epitopes. For all subsequent Western blot experiments, protein lysates were incubated for 15 min at room temperature before loading on the SDS-PAGE.

**
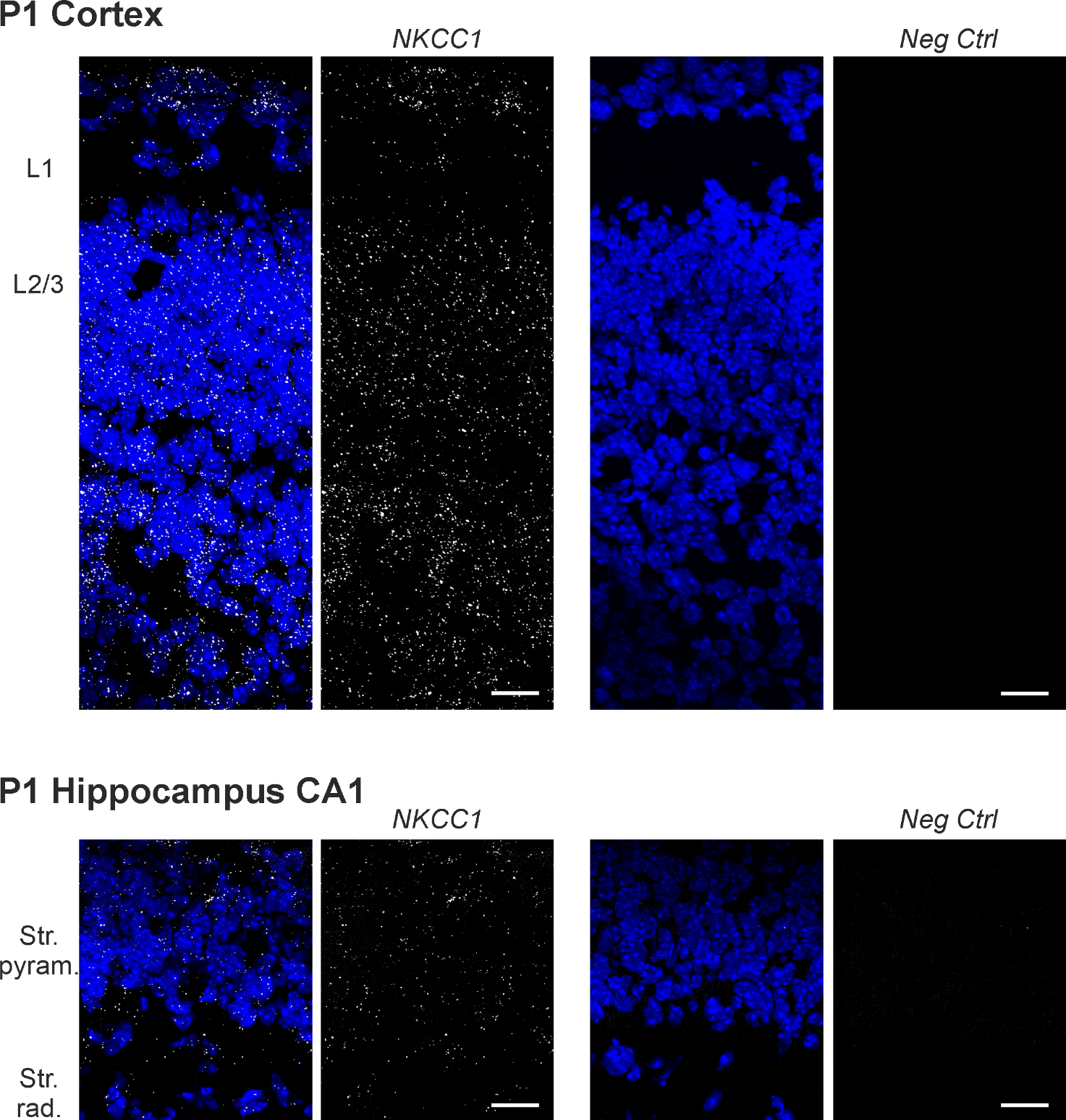
**

**Supplementary Figure 12: Extended data on NKCC1 mRNA expression in the P1 cortex and hippocampus shown by the ultrasensitive RNAscope *in situ* hybridization**

NKCC1 mRNA expression in the P1 somatosensory cortex and hippocampal CA1. Negative control probes show no detectable signal. *Scale bars 20 μm*

**
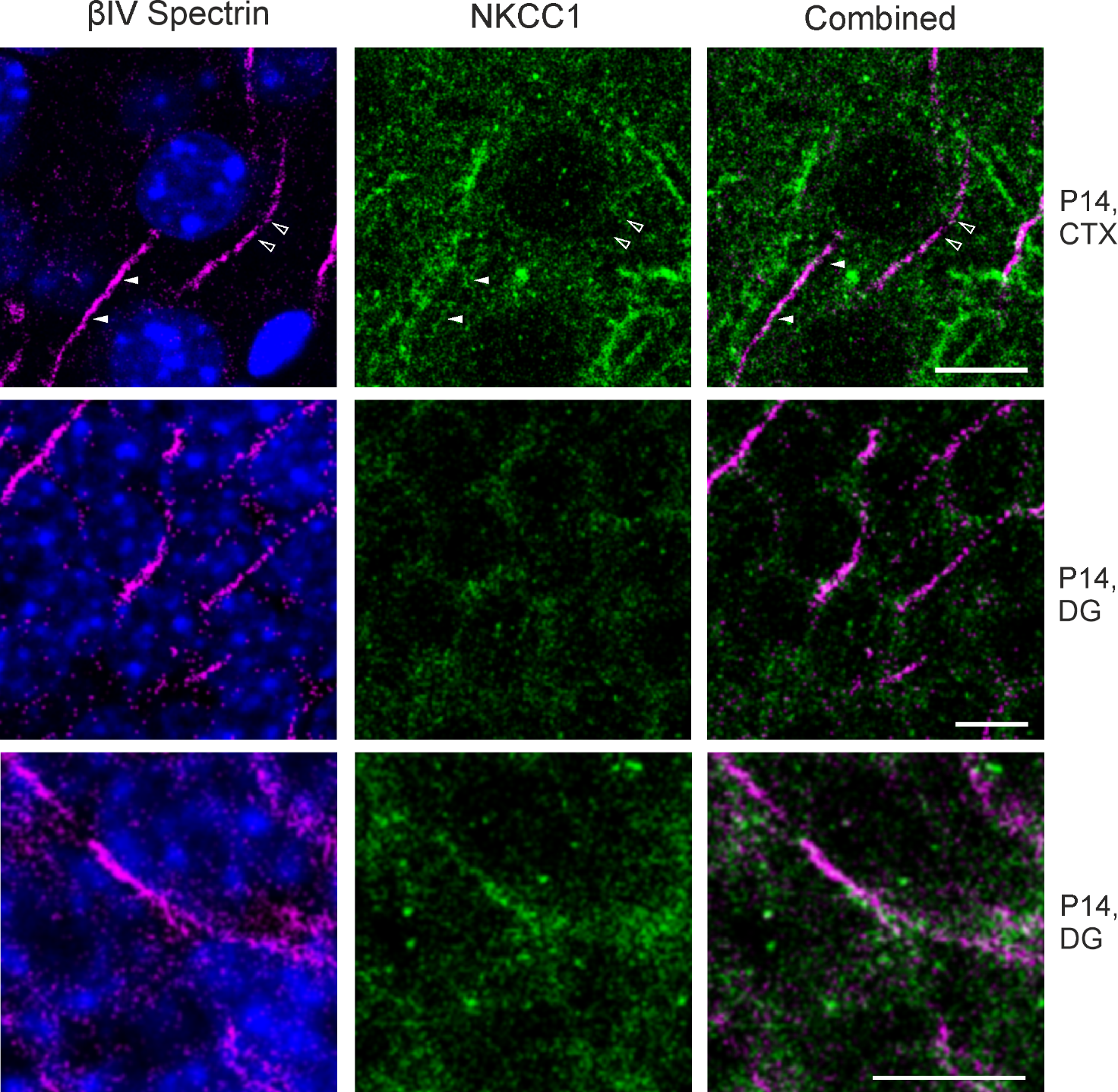
Supplementary Figure 13: NKCC1 IR is ambiguous in neuronal AIS, indicating that present techniques were not sensitive enough to resolve the NKCC1 expression in subcellular neuronal compartments**

Filled arrowheads pointing to plausible weak NKCC1 IR colocalizing with the AIS marker βIV spectrin. Empty arrowheads pointing to absence of such colocalization in other AIS. *Scale bar 10 μm*
